# Relative Abundance of Spermadhesin-1 in the Seminal Plasma of Young Nellore Bulls Is in Agreement with Reproductive Parameters

**DOI:** 10.3390/vetsci10100610

**Published:** 2023-10-07

**Authors:** Camilo José Ramírez-López, Edvaldo Barros, Pedro Marcus Pereira Vidigal, Denise Silva Okano, Juliana Nascimento Duarte Rodrigues, Lidiany Lopes Gomes, José Carlos Montes-Vergara, Victor Gerardo Petro Hernandez, Maria Cristina Baracat-Pereira, Simone Eliza Facioni Guimarães, José Domingos Guimarães

**Affiliations:** 1 Laboratory of Animal Reproduction, Department of Veterinary Medicine, Universidade Federal de Viçosa, Viçosa 36570-900, Brazil; deniseokano@gmail.com (D.S.O.); juliana.nascimento@ufv.br (J.N.D.R.); lidianyveterinaria@gmail.com (L.L.G.); jdguima@ufv.br (J.D.G.); 2 Department of Animal Science, Universidad de Córdoba, Monteria 230002, Colombia; josemontesv@correo.unicordoba.edu.co; 3 Laboratory of Proteomics and Protein Biochemistry, Department of Biochemistry and Molecular Biology, Universidade Federal de Viçosa, Viçosa 36570-900, Brazil; baracat@ufv.br; 4 Nucleus for Analysis of Biomolecules, Universidade Federal de Viçosa, Viçosa 36570-900, Brazil; edvaldo.barros@ufv.br (E.B.); pedro.vidigal@ufv.br (P.M.P.V.); 5 Department of Veterinary Medicine, Universidad de Santander, Valledupar 200001, Colombia; vic.petro@mail.udes.edu.co; 6 Laboratory of Animal Biotechnology, Department of Animal Science, Universidade Federal de Viçosa, Viçosa 36570-900, Brazil; sfacioni@ufv.br

**Keywords:** proteomic, reproduction, andrology, bovidae, livestock

## Abstract

**Simple Summary:**

Seminal plasma is a complex secretion that is produced in various organs of the male reproductive system. It is rich in proteins that play an essential role in the proper functioning of spermatozoa. Proteins found in seminal plasma are involved in processes such as sperm capacitation, the acrosome reaction, and providing antioxidant and immunological protection to spermatozoa. Differences in seminal plasma protein abundance may be linked to changes in the reproductive parameters of bulls. To investigate this possible association, we analyzed the proteomic profile of the seminal plasma of young Nellore bulls and its relationship with the results of the Breeding Soundness Evaluation (BSE). This study aimed to establish a link between the differential protein profile and the phenotypic characteristics of the semen of young bulls approved and not approved for reproduction.

**Abstract:**

This study aimed to evaluate the proteomic profile of seminal plasma from young Nellore bulls. We used 20 bulls aged between 19.8 and 22.7 months, divided into two groups according to the results of the Breeding Soundness Evaluation (BSE): approved (FIT *n* = 10) and not approved (UNFIT *n* = 10). The scrotal perimeter was measured and a semen collection was performed through electroejaculation. The percentage of sperm motility, mass motility, and sperm vigor were calculated using conventional microscopy, and the percentage of sperm abnormalities was calculated using phase-contrast microscopy of all ejaculates. Seminal plasma was separated from spermatozoa using centrifugation and processed for proteomic analysis by LC-MS/MS. Seminal plasma proteins were identified using MASCOT Daemon software v.2.4.0 and label-free quantification analysis was carried out by SCAFFOLD Q+ software v.4.0 using the Exponentially Modified Protein Abundance Index (emPAI) method. Functional classification of proteins was performed based on their genetic ontology terms using KOG. Functional cluster analysis was performed on DAVID. There were no differences in scrotal perimeter and physical semen characteristics between FIT and UNFIT groups of bulls. The percentage of sperm abnormalities was higher (*p* < 0.05) in the UNFIT group of bulls. A total of 297 proteins were identified for the two groups. There were a total of 11 differentially abundant proteins (*p* < 0.05), two of them more abundant in FIT bulls (Spermadhesin-1 and Ig gamma-1 chain C region) and nine in UNFIT bulls (Vasoactive intestinal peptide, Metalloproteinase inhibitor 2, Ig lambda-1 chain C regions, Protein FAM3C, Hemoglobin beta, Seminal ribonuclease, Spermadhesin 2, Seminal plasma protein BSP-30kDa, and Spermadhesin Z13). Spermadhesin-1 was the protein with the highest relative abundance (36.7%) in the seminal plasma among all bulls, corresponding to 47.7% for the FIT bulls and 25,7% for the UNFIT bulls. Posttranslational modification, protein turnover, and chaperones were the functional categories with the highest number of classified proteins. Protein functional annotation clusters were related to Phospholipid efflux, ATP binding, and chaperonin-containing T-complex. The differentially abundant proteins in the group of FIT bulls were related to sperm capacitation and protection against reactive species of oxygen. In contrast, differentially expressed proteins in the group of UNFIT bulls were related to motility inhibition, intramembrane cholesterol removal and oxidative stress. In conclusion, the proteomic profile of the seminal plasma of FIT bulls presents proteins with participation in several biological processes favorable to fertilization, while the proteins of the seminal plasma of UNFIT bulls indicate a series of alterations that can compromise the fertilizing capacity of the spermatozoa. In addition, the relative abundance of spermadhesin-1 found in the seminal plasma of young Nellore bulls could be studied as a reproductive parameter for selection.

## 1. Introduction

Brazil is a remarkable producer in beef cattle farming, ranking as the second largest beef exporter globally [1]. This economic activity owes its success to several factors, including Brazil’s expansive land, natural and improved grassland utilization, and the breed makeup of their cattle, comprised mainly of Nellore cattle and their hybrids [2]. These characteristics give Brazil a competitive edge in the meat production industry compared to other countries.

Nellore cattle are known for their remarkable ability to adapt to different environmental conditions, high rusticity, and resistance to diseases, particularly those caused by ectoparasites. Additionally, rigorous genetic improvement programs aimed at selecting traits of high commercial value, such as sexual precocity and accelerated weight gain, have facilitated shorter production cycles, thereby increasing the intensity and efficiency of meat production systems [3,4].

Scientific advancements in recent decades have led to the widespread use of various reproductive biotechnologies, accelerating genetic progress [5]. However, a large percentage of beef cattle breeders continue to rely on natural mating systems as a reproductive management strategy for their herds [6]. Regardless of the adopted reproductive strategy, the reproductive performance of the bulls is an outstanding characteristic, as a single sire is responsible for mating with multiple cows [7]. Thus, the bulls’ reproductive capacity directly impacts the cows’ pregnancy rates and, consequently, the farm’s overall productivity.

Breeding Soundness Evaluation (BSE) is a fundamental procedure for assessing the factors that influence the reproductive performance of bulls. It involves evaluating clinical aspects, genital organ assessment, semen phenotypic characteristics, and sperm morphology [8]. When all traits evaluated during BSE are within normal physiological standards, bulls receive approval to be used as breeders (FIT); otherwise, they are not approved (UNFIT). Depending on the potential reversibility of the causes that led to the non-approval, bulls may be re-evaluated 60 days after the BSE.

Despite the rigor of BSE, certain conditions related to the properties of the seminal microenvironment can compromise sperm quality and, consequently, reduce the reproductive capacity of bulls [9]. These conditions are not detected by conventional sperm analysis. These microenvironmental properties are associated with the composition of seminal plasma (SP), which is the liquid component of semen responsible for transporting and protecting ejaculated spermatozoa [10].

SP is a mixture of secretions from the testicles, epididymis, and mainly the accessory sex glands, containing minerals, sugars, carbohydrates, and proteins [11]. As a transcriptionally silenced cell, many spermatozoa functions depend on the SP’s proteins to perform adequately [12]. Thus, SP proteins, through their binding to the sperm plasma membrane, participate in sperm maturation, sperm motility, metabolism, protection, capacitation, acrosome reaction, and the oocyte–sperm interaction [13,14,15]. Proteomic analysis of SP allows for identifying and quantifying proteins present in this fluid. This analysis provides valuable information about sperm quality, the function and health of the genital tract in which spermatozoa mature, and the reproductive potential of bulls. Furthermore, understanding the function of proteins present in SP can reveal specific markers associated with fertility and semen quality, enabling a more accurate and efficient selection of sires [16,17].

Therefore, the objective of the present study was to determine the SP proteomic profile of young Nellore bulls classified as FIT and UNFIT for reproduction and identify differentially expressed proteins that might relate to the morphological characteristics of the sperm.

## 2. Materials and Methods

### 2.1. Ethics Approval

This study was approved by the Ethics Committee for the Use of Animals of the Universidade Federal de Viçosa (proc. n 28/2017). All methods were carried out under relevant guidelines and regulations, and all methods were reported following the ARRIVE guidelines (https://arriveguidelines.org, accessed on 17 July 2017).

### 2.2. Selection of Animals

Twenty Nellore bulls, aged between 19.8 and 22.7 months, belonging to a bovine herd genetically selected for having the best conditions for weight gain, sexual precocity, and carcass quality, were equally distributed into two groups based on their reproductive characteristics. The bulls come from a Nellore male production farm in the city of Aquidauana, State of Mato Grosso do Sul, in Brazil (19°48′53.3124″ S, 55°40′8.2704″ W), where the animals are grown in the field and fed on *Brachiaria brizantha* grass and water ad libitum. The FIT group (Group 1, consisting of 10 bulls approved for reproduction) and the UNFIT group (Group 2, consisting of 10 bulls not approved for reproduction) were determined according to criteria established by the Brazilian College of Animal Reproduction (Colégio Brasileiro de Reprodução Animal—CBRA) [18]. These criteria establish a minimum requirement for bull approval for reproduction, including 50% motility and 70% normal sperm.

All bulls underwent Breeding Soundness Evaluation (BSE), which consisted of examining the internal reproductive organs, such as prostate and seminal vesicles, through transrectal palpation and assessment of the prepuce, penis, testes, and epididymis, observing the shape, size, position, symmetry, and consistency of each organ. Subsequently, the scrotal perimeter was measured with scale tape at the largest region of the scrotum. Semen was collected by electroejaculation, and seminal physical characteristics and sperm morphology were assessed [19].

Semen phenotypic characteristics were evaluated subjectively by the same technician estimating mass motility (on a scale from 0 to 5, where 0 represented the absence of mass motility and 5 accented mass motility), sperm motility (percentage 0–100%, where 0% indicated no cells with movement and 100% indicated all cells with movement), and sperm vigor (on a scale from 0 to 5, where 0 represented no movement and 5 represented rapid and vigorous movements) in an aliquot of semen analyzed under conventional optical microscopy (CX31, Olympus, Tokyo, Japan) at 100× magnification, observing at least four microscopic fields in each sample. The results were expressed in the average of the fields as described by Okano et al. [20]. Additionally, the volume (mL) and physical aspect (1: aqueous, 2: opalescent, 3: milky, 4: creamy) of each ejaculate were estimated.

For morphological analysis, a semen aliquot from the sampled bulls was diluted in 1 mL of formaldehyde-buffered saline [21]. Sperm defects were evaluated in wet preparation, between slide and coverslip at 1000× magnification under immersion, and assessed by phase contrast microscopy (BX41, Olympus, Tokyo, Japan). Four hundred cells were counted for each sample, and the percentage of sperm defects in the acrosome, head, midpiece, and tail was determined. Subsequently, three categories of defects were defined: major (defects related to impaired fertility), minor (other deviating forms of minor importance), and total defects (the sum of major and minor defects), according to Blom [22], as recommended by CBRA [18].

### 2.3. Seminal Plasma Preparation for Proteome Analysis

The ejaculate from each bull was centrifuged (Heraeus Multifuges X1R, Thermo Scientific, Waltham, MA, USA) at 10,000× *g* for 10 min to separate sperm from SP. The SP samples were recovered and stored in 0.5 mL straws in liquid nitrogen at −196 °C until processing. Subsequently, 0.5 mL of SP from each bull was thawed and filtered through a 0.22 µm mesh membrane, and the filtered SP was used in the downstream analysis [23].

### 2.4. Extraction and Quantification of Soluble Proteins

Fractions containing 100 µL of filtered SP were precipitated by 600 µL of ice-cold solution acetone: ether was (1:1) added to trichloroacetic acid (5%, *w*/*v*) and 1 mM dithiothreitol (DTT; Sigma-Aldrich, Burlington, NJ, USA) and resuspended in 250 µL of solubilization solution (7 M urea, 2 M thiourea, 4% (*w*/*v*) CHAPS (3-3-cholamidopropyl dimethylammonium-1-propanesulfonate; Sigma-Aldrich, Burlington, NJ, USA), and 40 mM DTT)). Then, SP proteins were quantified using the Bradford method after calibrating a curve from known concentrations of bovine serum albumin [24]. 

### 2.5. SDS-PAGE

An aliquot of each sample containing 25 µg of soluble proteins was analyzed by one-dimensional electrophoresis (SDS-PAGE) to verify the quality of the protein extraction and quantification processes. SDS-PAGE (separation gel: 14% T, 3.0% C; stacking gel: 5.1% T, 2.6% C) was performed at 80 V for 15 min, following 60 V for 4 h, using mini-gel equipment (Mini-PROTEAN Tetra Cell, Bio-Rad, Hercules, CA, USA) and a molecular weight marker (Broad Range, Bio-Rad, Hercules, CA, USA). Gels were stained with Coomassie Brilliant Blue R-250 (Bio-Rad, Hercules, CA, USA) for 2 h and destained for 10 h in a solution of methanol 25% (*v*/*v*; Sigma-Aldrich, Burlington, NJ, USA) and acetic acid 7.5% (*v*/*v;* Sigma-Aldrich, Burlington, NJ, USA) [25].

### 2.6. Sample Pooling

After assessing the prepared protein samples, fractions containing 60 µg of protein from each bull sample were added to a new tube to pool two groups of animals according to reproductive characteristics (FIT and UNFIT for reproduction). Subsequently, five replicates of each pool containing 50 µg of proteins were subjected to electrophoresis for approximately one centimeter of resolution in SDS-PAGE 12.5% gel (separation gel: 12.5% T, 3.3% C; stacking gel: 5.1% T, 2.6% C). Then, gels were cut into two parts and placed in a fixative solution (40% (*v*/*v*) methanol and 5% (*v*/*v*) acetic acid) for 2 h [26]. Part of the gel was stained using the Coomassie Brilliant Blue R-250 method (Bio-Rad, Hercules, CA, USA) for 2 h, and the other part remained in a 5% (*v*/*v*) acetic acid solution for further enzymatic digestion.

### 2.7. Gel Protein Digestion

All parts of the gels from the five replicates were excised individually, pooled, cut into smaller fragments, and transferred to clean microtubes. The dye was removed from the gels after three washes in 200 µL of 50% (*v*/*v*) acetonitrile (ACN; Sigma-Aldrich, Burlington, NJ, USA) and 25 mM ammonium bicarbonate (AMBIC, pH 8.0; Sigma-Aldrich, Burlington, NJ, USA).

Gel fragments were dehydrated after two incubations with 200 µL of 100% ACN for 5 min and dried using a vacuum concentration system (Concentrator Plus AG—22331, Eppendorf, Hamburg, Germany). The proteins present in the gel fragments were reduced in 100 uL of 65 mM DTT in 100 mM AMBIC at 56 °C for 30 min. Then, proteins were alkylated with 100 µL of 200 mM iodoacetamide and 100 mM AMBIC at room temperature for 30 min in the dark. Subsequently, gel pieces were washed, hydrated, and dehydrated twice, using AMBIC and ACN, sequentially. Finally, they were dried in a vacuum concentration system.

Dried gel fragments were rehydrated with trypsin solution (Promega, Madson, WI, USA) at a final concentration of 25 ng/µL in activation solution (40 mM AMBIC and 10% (*v*/*v*) ACN). After 45 min in an ice bath, tubes containing the gel pieces were added to 100 µL of the activation solution without trypsin. The tubes were placed in a 37 °C water bath for 22 h. After enzymatic digestion, the gels were sonicated for 10 min, vortexed for 20 s, and the solutions were removed to clean microtubes. Next, 200 µL of the recovery solution (5% (*v*/*v*) formic acid in 50% (*v*/*v*) ACN) was added to the remaining gel fragments. Each microtube containing the gel fragments and recovery solution was vortexed for 20 s, kept at room temperature for 15 min, and sonicated for 2 min. The solution was removed and added to the previously reserved solution in the clean tube. This step was repeated twice, and the solution was transferred to clean microtubes. Solutions containing the tryptic peptides were concentrated in a vacuum centrifuge system [27]. After vacuum drying, 10 µL of 0.1% (*v*/*v*) trifluoroacetic acid (TFA; Sigma-Aldrich, Burlington, NJ, USA) solution with ultrapure water was added to each microtube. Then, the tryptic peptides were desalted using C18 reversed-phase micro columns (Millipore, Burlington, NJ, USA) and eluted in a 50% ACN solution, acidified with 0.1% (*v*/*v*) TFA.

### 2.8. LC-MS/MS

The tryptic peptides from each replicate were solubilized in 20 µL of 0.1% (*v*/*v*) aqueous formic acid solution (LC-MS purity grade, Sigma-Aldrich, Burlington, NJ, USA) and placed in appropriate tubes for application to the nano LC-MS/MS system. Analysis of 1 μL of the sample was carried out using the UPLC nanoAcquity system (Waters, Milford, MA, USA), containing a nanoAcquity UPLC^®^ 2G-V/MTrap 5 µm Symmetry^®^ C18 180 µm × 20 mm trap column, at a flow rate of 7 µL/min, for 3 min. Peptides were separated using a 1.7 µm BEH130 100 µm × 100 mm nanoAcquity UPLC^®^ column, operating at a flow rate of 0.3 µL/min. The two solvents used as a mobile phase of the chromatographic process were ultrapure water acidified with 0.1% (*v*/*v*) formic acid (solvent A) and pure ACN acidified with 0.1% (*v*/*v*) formic acid (solvent B). Chromatographic separation took place according to the following schedule: 2% B for 1 min; gradient from 2 to 30% B for 299 min; gradient from 30 to 85% B for 5 min; maintenance at 85% B for 5 min; gradient from 85 to 2% B for 5 min; and maintenance at 2% B for 5 min, totaling 320 min of chromatographic analysis.

The eluted peptides were automatically injected into a MAXIS 3G model mass spectrometer (Bruker Daltonics, Billerica, MA, USA), operating online with a CaptiveSpray ionization source. Peptide analysis was performed by an appropriate method (IE_GCF_01-02-2017), with a drying gas flow of 3 L/min, ionization source temperature of 150 °C, and transmission voltage of 2 kV. The raw data were converted into a list of masses in Mascot Generic Format (MGF) and compared to a reference database.

### 2.9. Protein Identification

By using a Mascot Daemon version 2.4.0 (Matrix Science, London, UK), the MGF files were compared to the reference database that was a set of canonical protein sequences from the Bovidae family (Taxonomy ID 9895), available at the UniProt Knowledgebase database (UniProtKb; https://www.uniprot.org/; accessed on 28 March 2018). The search parameters used for peptide identification were the enzymatic digestion by trypsin with one missed cleavage, cysteine carbamidomethylation as a fixed modification, and methionine oxidation as a variable modification. The error tolerance allowed for the acquired data was 30 ppm for the parental ion and 0.6 Da for the fragments, with the ion charge varying between +2 and +4.

The Mascot Daemon results were validated by Scaffold Q+ version 4.0 (Proteome Software Inc., Portland, OR, USA). The identified peptides were validated by the Peptide Prophet algorithm [28]. The identified proteins were validated by the Protein Prophet algorithm [29] according to the following criteria: the probability threshold for identification was 0.95, the false discovery rate was less or equal to 1%, and the minimum number of peptides for protein identification was 2. In addition, we considered only proteins identified in at least two replicates.

### 2.10. Functional Classification of Proteins

The functional analysis used Gene Ontology (GO) terms attributed to the identified proteins available in the UniProtKB database. Functional enrichment analysis of GO terms was performed by the Database for Annotation, Visualization, and Integrated Discovery platform (DAVID; https://david.ncifcrf.gov/home.jsp; accessed on 11 November 2022) [30]. Identified proteins were classified by the RPSBLAST tool of BLAST version 2.13.0 in categories of the EuKaryotic Orthologous Groups (KOG) database, using an E-value threshold of 1 × 10^−10^ for selecting the significant alignments [31,32].

### 2.11. Quantitative Analysis of Identified Proteins

The label-free quantitative analysis considered only proteins identified in at least three technical replicates of each treatment. The abundance of selected proteins was estimated by the Exponentially Modified Protein Abundance Index (emPAI) using the Scaffold Q+ software version 4.0 [33]. Proteins with missing data were filtered out and not considered for differential expression analysis. Differentially abundant proteins (DAP) were identified by the msmsTests package using R version 4.2.2 (https://cran.r-project.org/, accessed on 29 March 2018) [34]. Tests for DAP identification used two models: one full model corresponding to the alternative hypothesis (UNFIT group) and another nested model corresponding to the null hypothesis (FIT group). The calculated *p*-values were corrected using the Benjamini–Hochberg procedure, and DAP proteins were those with FDR < 0.05.

### 2.12. Analysis of Protein Interaction Networks

The proteins that exhibited differential abundances between the groups were analyzed using the STRING 11.5 database (http://www.string-db.org, accessed on 11 November 2022) [35]. This analysis was based on predictions collected from various sources, including direct (physical) or indirect (functional) interactions, integrating evidence from multiple sources such as genomic context, large-scale experiments, co-expression, and data obtained from research publications.

### 2.13. Phenotypic Data Analysis

Differences in semen phenotypic characteristics among the experimental groups were assessed by analysis of variance (ANOVA) using SAEG version 9.1 and a significance threshold of 5% [36]. Pearson’s correlation coefficients were used to determine the relationship between the characteristics studied, at a significance level of 5%.

## 3. Results

### 3.1. Spermogram of Bulls Classified as FIT and UNFIT for Reproduction

The semen phenotypic characteristics mass motility (MM), sperm motility (Mot), and sperm vigor (Vig) did not differ (*p* < 0.05) between the approved (FIT) and not approved (UNFIT) bull groups for reproduction. However, the percentage of sperm defects (Morp) was significantly lower in the FIT group when compared to the UNFIT one (Table 1). Furthermore, no differences were observed between the bulls’ scrotal perimeter (PE) average values.

### 3.2. Proteomic Characterization of Seminal Plasma from Nellore Bulls

**Figure 1:**
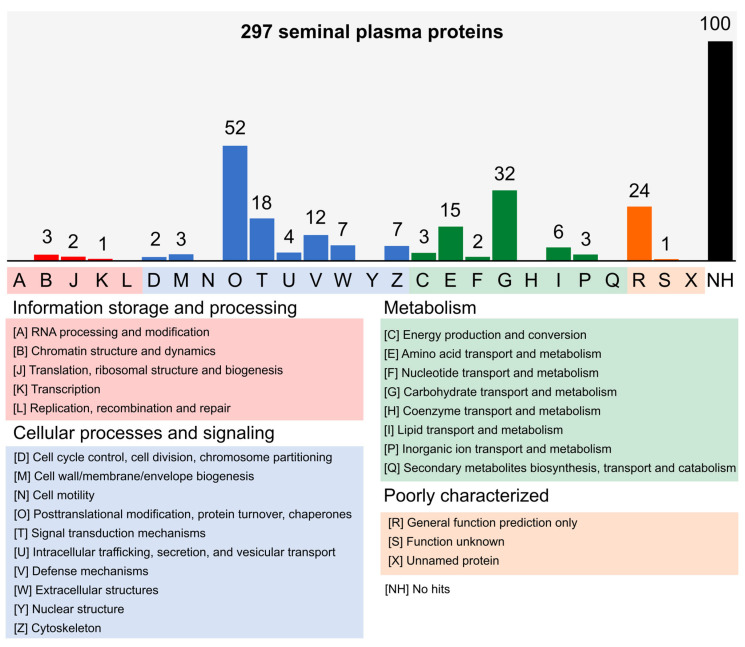
Functional classification of seminal plasma proteins from Nellore bulls. The sequence similarity searches classified 197 proteins into four main groups of the KOG database. One hundred proteins did not show significant hits [NH] and were not classified. The twenty-six letters from [A] to [Z] represent the functional categories of the KOG database.

The LC-MS data analysis identified 297 proteins in total for the SP of bulls classified as FIT and UNFIT for reproduction (Supplementary Table S1). Based on the functional categories of the KOG database, those with the highest number of annotated proteins were posttranslational modification, protein turnover, and chaperones [O]; carbohydrate transport and metabolism [G]; and general function prediction [R] (Figure 1 and Supplementary Table S2).

Other functional categories that must be highlighted due to their importance in sperm physiology are signal transduction mechanisms [T]; amino acid transport and metabolism [E]; and defense mechanisms [V] (Figure 1). It should be noted that these proteins were identified in both FIT and UNFIT bulls, showing varying intensities, except for the following proteins: epididymal sperm binding protein 1 (UniProtKB ID E1B9P4); SERPIN domain-containing protein (UniProtKB ID L8I2G8); ARSG protein (UniProtKB ID A6QLR7); protocadherin fat 2 (UniProtKB ID L8IHR1); insulin-like growth factor II (UniProtKB P07456); leucine-rich alpha-2-glycoprotein 1 (UniProtKB Q2KIF2); vitamin D-binding protein (UniProtKB ID Q3MHN5); GDP-fucose protein O-fucosyltransferase 2 (UniProtKB ID Q7YRE5); and gastrin-releasing peptide (UniProtKB ID Q863C3). Proteins E1B9P4 and L8I2G8 were found only in FIT bulls, while the others were exclusive to UNFIT bulls (Supplementary Table S1).

Spermadhesin-1 represented 47.7% of the total abundance of proteins identified in the FIT experimental group and 25.7% of the UNFIT group, followed by seminal plasma protein PDC-109 (2.3% of FIT and 8.2% of UNFIT) and serine protease inhibitor clade E member 2 (3.9% in both groups). Relative quantification also revealed that 19 proteins were responsible for 70.5% of the total estimated protein abundance for the FIT group and 72.1% for the UNFIT group (Table 2), showing that, of the vast majority of proteins identified in our study, 278 (Supplementary Table S1) were low-abundance proteins.

The functional enrichment analysis distributed the SP proteins in 48 clusters related to relevant biological mechanisms, pathways, molecules, and cellular structures. The main functional annotation of the clusters with the highest enrichment score based on the GOs of biological processes (BP), molecular functions (MF), and cellular components (CC) are related, respectively, to events involving sperm capacitation, functional maintenance of proteins, and components for better functionality of proteins regarding reproduction (Table 3).

### 3.3. Quantitative Proteomics of Seminal Plasma from FIT and UNFIT Bulls for Reproduction

**Figure 2:**
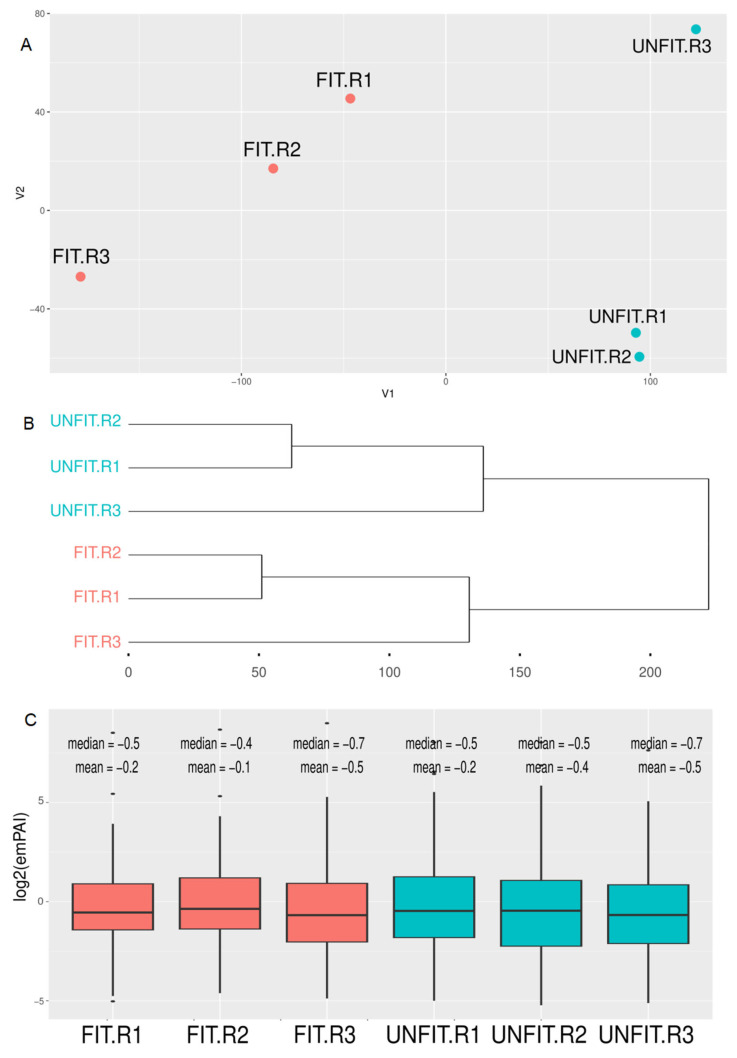
Principal component analysis (PCA) and clustering of the label-free quantification data. (**A**) The PCA shows the separation between the technical replicates of the FIT and UNFIT bulls. (**B**) The quantification data clustered using the UPGMA (unweighted pair group method with arithmetic mean) method also shows this separation. (**C**) The boxplots show the dispersion of quantification data and the respective mean and median values of the replicates.

**Figure 3:**
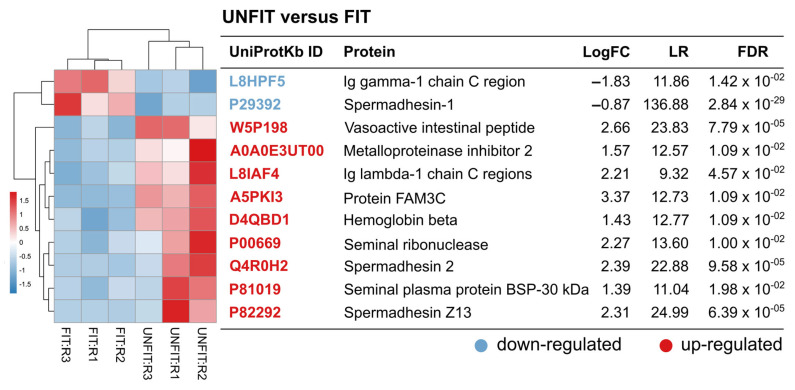
Differentially abundant proteins (DAP) in seminal plasma (SP) of young Nellore bulls classified as FIT and UNFIT for reproduction. The label-free quantification analysis identified 11 DAP in the SP of FIT and UNFIT bull groups. The heatmap shows the distribution of the DAP for the technical replicates. The colors represent the emPAI values submitted to the row scale: red squares indicate high abundance and blue squares indicate low one. LogFC: log-transformed fold-change. LR: likelihood ratio. FDR: *p*-values adjusted with FDR control by the Benjamini–Hochberg method.

**Figure 4:**
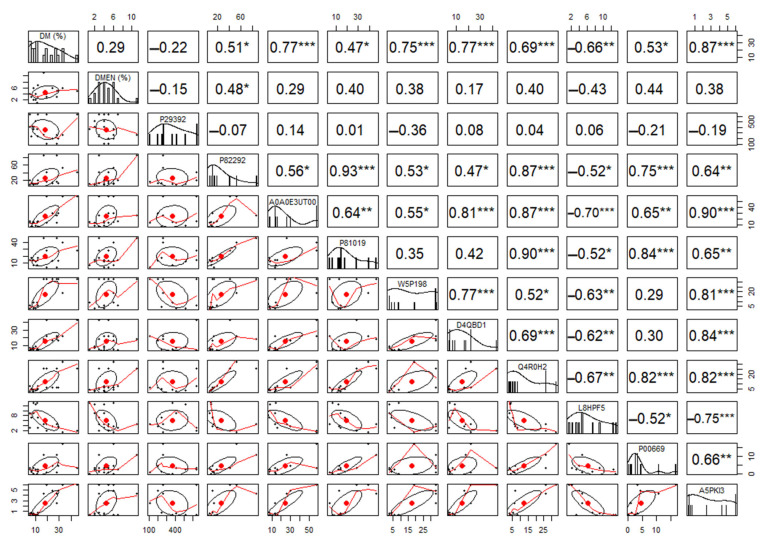
Analysis of Pearson’s correlation between the percentage of major and minor sperm defects with the differentially abundant proteins (DAP) in the seminal plasma (SP) of Nellore bulls classified as FIT and UNFIT for reproduction. DM: major sperm defects; DMEN: minor sperm defects. DAP: P29392: Spermadhesin 1; P82292: Spermadhesin Z13; A0A0E3UT00: metalloproteinase inhibitor 2; P81019: seminal plasma protein BSP-30 kDa; W5P198: vasoactive intestinal peptide; D4QBD1: hemoglobin beta; Q4R0H2: Spermadhesin 2; L8HPF5: Ig gamma-1 chain C region; P00669: seminal ribonuclease; A5PKI3: protein FAM3C. * *p* < 0.05; ** *p* < 0.01; *** *p* < 0.001.

Principal component and clustering analysis of label-free quantification data showed a clear separation between experimental groups of FIT and UNFIT bulls for reproduction (Figure 2). The comparison between the analyzed SP proteomes identified 11 DAP (FDR < 0.05), with two more abundant proteins in the FIT group (Spermadhesin-1 and Ig gamma-1 chain C region) and nine proteins in the UNFIT group (Figure 3). Pearson’s correlation analysis showed that the two proteins with the highest abundance in the group of FIT bulls presented medium and moderate negative correlations with the percentage of major and minor sperm defects, while three of the nine proteins with low abundance, namely, Protein FAM3C, tissue inhibitor of metalloproteinase-2 (TIMP-2), and Vasoactive intestinal peptide (VIP), showed strong and positive correlations with the variable percentage of major sperm defects in the group of bulls classified as UNFIT. No significant correlations were observed between Ig lambda-1 chain C and the percentage of sperm defects. Although the mean abundance value was higher in the UNFIT group, there was a high standard deviation between the results obtained in replicates, both for FIT and UNFIT (Figure 4).

**Figure 5:**
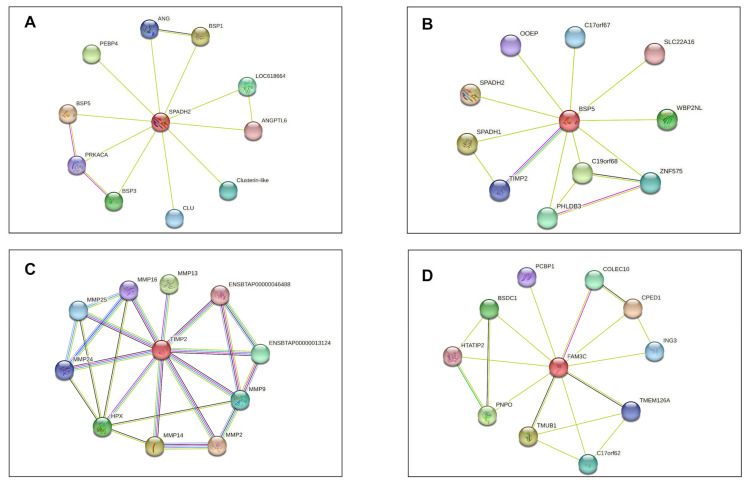
Protein–protein interaction networks from the seminal plasma of Nellore bulls. The networks were determined using STRING v.11.5 (https://string-db.org/, accessed on 11 November 2022) and exclusively include proteins differentially expressed in the group of bulls classified as UNFIT for reproduction. (**A**) SPADH2: Spermadhesin Z13; CLU: Clusterin; BSP1: binder sperm protein 1; BSP3: binder sperm protein 3; (**B**) BSP5: seminal plasma protein BSP-30 kDa; (**C**) TIMP 2: metalloproteinase inhibitor 2; (**D**) FAM3C: protein FAM3C; TMUB1: transmembrane and ubiquitin-like domain-containing protein 1; TMEM126A: *Bos taurus* transmembrane protein 126A; PNPO: Pyridoxine-5; CPED1: cadherin-like.

Based on in silico analysis of protein–protein interaction networks of DAPs, Spermadhesin Z13 (SPADH2) interacts with Clusterin (CLU) and members of the Binder Sperm Proteins family (BSP1, BSP3, and BSP5). On the other hand, the seminal plasma protein BSP-30 kDa (BSP5) interacts with Spermadhesin-1 (SPADH1) and Tissue Inhibitor Metalloproteinase 2 (TIMP2). TIMP2, in turn, interacts with several types of metalloproteinase. Protein FAM3C exhibits associations with transmembrane and ubiquitin-like domain-containing protein 1 (TMUB1), *Bos taurus* transmembrane protein 126A (TMEM126A), Pyridoxine-5 (PNPO) and cadherin-like (CPED1) (Figure 5).

## 4. Discussion

In the current study, we used young Nellore bulls classified as approved (FIT) and not approved (UNFIT) for reproduction by Breeding Soundness Evaluation (BSE). They were used as models to investigate differences in the seminal plasma (SP) proteomic profile of bulls with low and high percentages of sperm pathologies. The lack of variation (*p* < 0.05) of the evaluated phenotypic characteristics of the semen of FIT and UNFIT bulls, which were mass motility, sperm motility, sperm vigor and bulls’ scrotal perimeter average values, is probably due to genetic improvement program selection criteria. However, the percentage of sperm defects (Morp) was significantly lower in the FIT group when compared to the UNFIT group, confirming the excellence of the material (animals) selected to investigate differences in the proteomic profile of SP from bulls with low and high percentages of spermatic pathologies. In general aspects, our study found that the bulls had higher genetic values than the average young *Bos indicus* bull population.

It should be noted that the animals participating in this work, as they belong to a bovine herd genetically selected from a Nellore male production farm, have the best selection and maintenance conditions, and all methodologies employed followed the strictest standards of care for the animals. The electroejaculation method of semen collection and the evaluation of semen phenotypic characteristics followed the pattern routinely used on the farm, and were equally performed for all animals.

Molecular studies have emerged as crucial tools for comprehending andrological aspects that conventional assessments of a semen phenotype may not fully elucidate. Among these, proteomics has proven to be particularly promising, offering a deeper insight into reproductive events through the differential identification of proteins within groups of animals sharing similar characteristics. While the majority of such studies traditionally focus on adult bulls, the elevated costs associated with maintaining animals of high genetic value within breeding farms have sparked a growing interest in expediting the evaluation process, allowing for the rapid identification of UNFIT young bulls within herds. Consequently, there is a pressing need for a more comprehensive examination and understanding of the reproductive aspects specifically related to young bulls. These investigations can ultimately enhance the efficiency of animal breeding by facilitating more informed decisions and management strategies in the context of assisted reproduction.

This proteomic study employed the LC-MS/MS methodology, a versatile and powerful technique that offers the necessary sensitivity, precision, and identification capacity to study low-abundance proteins, as demonstrated by our results, where the abundance of 278 proteins corresponds to only about 28–30% of the total of 297 identified proteins. Low-abundance proteins often play crucial specific and regulatory roles in biological processes. They may be involved in signaling pathways, gene regulation, responses to external stimuli, and other important processes.

The functional classification profile of the identified proteins for the semen of FIT and UNFIT bulls highlights the integral role of SP in regulating and maintaining sperm function and protein quality. The SP plays a vital role in essential aspects of sperm biology, such as maturation, capacitation, motility, and acrosomal reaction [37], which was evidenced by the principal annotations obtained in the analysis of functional clusters and by the high scores obtained. The category named “Posttranslational modification, protein turnover, chaperones” in the KOG database is that with the highest number of annotated proteins. This was expected because SP constitutes the extracellular matrix of mature spermatozoa, whose protein transduction capacity is silenced during spermatogenesis, depending on the functions of SP proteins and their posttranslational modifications to carry out different processes [38,39].

In our study, addressing proteomics of SP in young Nellore bulls, Spermadhesin-1 constituted the protein identified with the greatest relative abundance, although Binder Sperm Proteins (BSPs) are reported to represent an abundance of about 60% in SP proteins for adult bulls [40]. Spermadhesin-1 is a protein secreted by the secretory cells of the seminal gland in Nellore bulls and plays several vital roles in reproductive processes [41]. For this purpose, Spermadhesin-1 can interact with the spermatozoa membrane, promoting sperm motility and protecting the spermatozoa from oxidative damage. Moreover, it binds to cervical mucus, facilitating the smooth progression of spermatozoa through the female reproductive tract. Spermadhesin-1′s crucial role extends to modulating the immune response in the reproductive tract, creating a favorable environment for sperm survival and fertilization. It achieves this by interacting with glycoproteins on the surface of immune cells, inhibiting their ability to recognize and bind to spermatozoa [42].

Furthermore, Spermadhesin-1 binds to plasma membrane phospholipids of spermatozoa stored in the ampullae of the vas deferens prior to ejaculation, which leads to a reduction in mitochondrial metabolism. This action protects sperm cells from damage caused by lipid peroxidation of the membranes while also acting as a motility deactivation factor, thus prolonging the life of the sperm. Additionally, some studies suggest that Spermadhesin-1 remaining in the sperm membrane after sperm capacitation may act as primary zona pellucida binding proteins, playing a significant role in sperm–egg recognition during fertilization [43,44,45,46]. Relatively high abundances of Spermadhesin-1 in the SP of bulls has shown positive associations with sperm freezability and cryopreserved semen fertility [47,48]. In contrast, the low abundance of Spermadhesin-1, observed in the group of bulls classified as UNFIT, would be related to the reduction in the fertilizing capacity of spermatozoa.

Despite not finding a cause that explains that Spermadhesin-1 is the most abundant protein in the SP of young Nellore bulls, based on its multiple functions and the results of the correlation analysis, we can hypothesize that relatively high levels of this protein indicate good sperm quality in young bulls. FIT bulls showed an abundance of Spermadhesin-1, on average, 78.3% higher than UNFIT bulls, with standard deviation values lower than 20%, for a three-replicate analysis. Considering the high total abundance of Spermadhesin-1 found in this work in the seminal plasma of young bulls, in general, the detection of higher abundances in young bulls could correspond to a differential characteristic for approved bulls (FIT), when compared to not approved ones (UNFIT). The early identification of not approved young bulls could allow the withdrawal of UNFIT bulls from the herd and reduce overall production costs.

Spermadhesin-1 is among the 19 proteins with an abundance greater than 1%, considering the total of 297 proteins identified for the seminal plasma of young Nellore bulls, both for FIT and UNFIT bull groups. This information is highly relevant because, despite the clear separation between the experimental groups FIT and UNFIT bulls, which was evidenced by PCA and clusterization analysis, 11 of the most abundant proteins in the seminal plasma correspond to differentially abundant proteins (DAP). When addressing the importance of finding a noninvasive biomarker to diagnose male factor infertility, also by proteomics, Kovac et al. [49] pointed out that these biological indicators (molecular markers) should identify problems at an early stage, be easily detectable, cost-effective, and accurate, as well as being objectively measured, evaluated, and compared. In this sense, these most abundant proteins found in this work, which are simultaneously DAPs, are candidates for studies aimed at identifying molecular markers. Most are directly involved in important reproduction events.

Contrary to Spermadhesin-1, Spermadhesin-2 and Spermadhesin Z13 showed higher relative abundance in the group of UNFIT bulls. Despite belonging to the same family, Spermadhesin-2 and Spermadhesin Z13 are phylogenetically and sequentially different from Spermadhesin-1, which could explain the difference in the observed abundance pattern in our study. Both proteins are involved in fertilization and are essential in binding sperm to the oviductal epithelium and modulating sperm function. However, there is limited evidence to suggest a relationship between high amounts of Spermadhesin-2 and Spermadhesin Z13 in SP with low fertility in bulls [50]. This relationship would be due to the inhibitory effect that large amounts of Spermadhesin-2 and Spermadhesin Z13 exert on sperm motility by interfering with the activation of Dynein ATPase. This enzyme acts in the hydrolysis of ATP in the midpiece microtubules [51]. Despite not being able to establish a direct relationship between Spermadhesin Z13 and a high percentage of sperm defects that could explain its high abundance in the group of UNFIT bulls, in silico analyses of protein–protein interactions show a link between Spermadhesin Z13 (SPADH2) and Clusterin (CLU).

Clusterin is a protein that protects against oxidative stress by helping neutralize free radicals and reduce oxidative damage to sperm cells. It also binds to seminal plasma components, stabilizing lipid and protein particles. However, it has been inversely associated with morphologically normal sperm populations, indicating poor semen quality in bulls [52].

Seminal plasma protein BSP-30 kDa (BSP 5) and seminal ribonuclease are proteins that have been widely studied concerning their roles in SP and bull fertility [52]. Both proteins have been identified as good phenotypic indicators of bulls with high fertility and freezability [53,54]. However, these findings contradict our results, since BSP 5 and SRN showed greater abundance in the group of bulls classified as UNFIT for reproduction and significant positive correlations with the percentage of major sperm defects, indicating that they are possibly related to the percentage of significant sperm defects. Exposure of sperm to BSP 5 in high concentrations for prolonged periods causes damage to the sperm membrane due to the excessive elimination of intramembrane compounds such as cholesterol and phospholipids [55].

Under normal conditions, BSP 5 interacts with spermatozoa during ejaculation, partially binding to plasma membrane cholesterol and phosphatidylcholine [56]. This way, it participates in processes such as spermatozoa binding to oviduct epithelium, sperm capacitation, and interaction between oocyte and spermatozoa [57]. However, Moura et al. [51] found a quadratic association between the amount of BSP 5 and bulls’ fertility, indicating that BSP 5 benefits semen adequately. Nevertheless, its excess harms fertility, reaffirming that activity of BSP 5 is concentration-dependent.

In turn, Seminal ribonuclease participates in sperm capacitation and protects spermatozoa due to its anti-spermatogenic, antitumor, and immunosuppressive activity. Despite its properties, Westfalewicz et al. [58] observed an increase in seminal ribonuclease abundance in warmer periods of the year when compared to colder periods; in the warmer periods, the seminal quality parameters of bulls were lower. They proposed that high environmental temperatures induce seminal ribonuclease aggregation in the form of oligomers, increasing the enzymatic and cytotoxic activity of the ejaculates and making SP a harmful environment for spermatozoa [59]. The city of Aquidauana, located in the state of Mato Grosso do Sul, Brazil, where our experiments were conducted, experiences higher climatic temperatures compared to Olecko, Poland, where Westfalewicz et al. [58] conducted their experiment. Therefore, the temperature effect on seminal ribonuclease is expected to be more significant in the bulls of our experiment. However, further tests are necessary to confirm such an effect.

Another protein identified with greater abundance in the group of UNFIT bulls, showing strong positive correlations with major sperm defects, was Tissue Inhibitor Metalloproteinase 2 (TIMP-2), a semen constituent protein that modulates the activity of proteolytic enzymes such as matrix metalloproteinases (MMP) through the formation of inhibitory molecular complexes [60]. MMPs and TIMPs participate in tissue restructuring through extracellular matrix remodeling. Their functions are fundamental in semen liquefaction within the female reproductive system, invasion of trophoblast cells into the endometrium, and angiogenesis during embryo implantation [61].

Although other studies have identified TIMP-2 in the SP of bulls with high fertility and in human spermatozoa with a low degree of DNA fragmentation, the high abundance of TIMP-2 might also indicate testicular degeneration [53,62,63]. It is due to its participation in inflammatory processes, structural and functional changes in Sertoli cells, and the functioning of the blood–testis barrier, as suggested by Pereira et al. [64] and based on their results obtained from bulls subjected to testicular heat stroke. In fact, Newton et al. [65] observed that the level of TIMP-2 expression increases as the testicular temperature of bulls rises. Furthermore, Boe-Hansen et al. [10] also reported a positive correlation between a high abundance of TIMP-2 and a high percentage of sperm defects in the seminal plasma of young Brahman bulls.

In our study, TIMP-2 also showed a significant correlation with a high abundance of seminal ribonuclease. The high abundance of both proteins has been related to low sperm quality due to increased temperature, as mentioned above. Increased scrotal temperature can change the composition of seminal plasma due to direct changes in the testicular parenchyma or in the epididymis, or indirectly through changes in hormonal or neurological pathways that affect the composition of accessory fluids of the sex glands [66]. It is known that many of the sperm pathologies considered to be major defects are due to testicular diseases and alterations in spermatogenesis induced by factors such as heat stress [67]. Increased testicular temperature interrupts spermatogenesis at all stages, leading to an increase in morphologically abnormal sperm and a reduction in motility and sperm concentration due to apoptosis [68,69].

The Vasoactive intestinal peptide (VIP) is a neuropeptide identified in several tissues, such as testes, accessory sex glands, and nerve fibers of central and peripheral nervous systems, where it plays a role as a neurotransmitter and an active neuroendocrine substance [70,71]. In mammals and birds, VIP acts as a prolactin-releasing factor, a polypeptide hormone that participates in many biological functions, including reproduction [72].

In males, prolactin inhibits the hypothalamic secretion of GnRH and, consequently, inhibits the pulsatile release of FSH and LH in the pituitary gland, reducing testicular testosterone, resulting in alterations in spermatogenesis, poor sperm quality, and infertility [73]. Studies in roosters have shown the inhibitory effect of VIP on reproductive performance and sperm quality in young individuals due to VIP’s role in prolactin release [74]. These observations might partially explain our findings, in which VIP was more abundant in the UNFIT group and is possibly related to sperm defects. Pearson analysis showed a significant correlation between high VIP abundance and major sperm defects, in addition to also showing a relationship with high abundance of FAM3C and Hemoglobin beta.

FAM3C and Hemoglobin beta are proteins that play several roles in biological processes such as osteogenesis, regulation of energy metabolism, and oxygen transport [75,76]. In SP from bulls, both proteins are involved in alternative mechanisms of sperm protection against damage caused by lipid peroxidation occasioned by reactive oxygen species [57,77].

The reported protective mechanisms that FAM3C and Hemoglobin beta proteins exert are unclear. However, they seem to be related to the binding capacity of iron and copper ions, which, in large amounts, can trigger the formation of free radicals from cholesterol peroxidation of cytoplasmic membranes [78]. Also, free iron ions in SP can indirectly cause oxidative stress in sperm due to iron being used as a substrate by various pathogenic microorganisms, triggering immune reactions, infiltration of macrophages, and activating neutrophils capable of producing large amounts of reactive oxygen species [79,80,81].

The role of immunoglobulins G (IgG) in PS is a controversial topic in scientific literature. Some studies have linked the presence of IgG to the fusion of male and female gametes, as well as the regulation of the immune response in the male genital tract. However, other studies have associated the presence of IgG in SP with subfertility in men due to the binding of these molecules to antigens present on the sperm surface, causing agglutination and immobilization. This, in turn, prevents the penetration of cervical mucus and leads to failures in the acrosomal reaction [82,83].

Our study has identified IgGs as differentially abundant in both FIT and UNFIT bulls, which may add to the controversy. In particular, the Ig gamma-1 chain C region is DAP in FIT bulls. This is the constant region of the heavy chain of the IgG1 immunoglobulin and plays a key role in the effector activities of the antibody after binding to antigens. We found significant negative correlations between this protein and the percentage of major sperm defects, as well as the high abundance of other proteins, such as the FAM3C protein, hemoglobin beta, and TMP-2.

On the other hand, the Ig lambda-1 chain C regions protein is differentially abundant in UNFIT bulls. This protein is a part of the structure of a lambda-1 immunoglobulin light chain, which is a component of antibodies produced by the immune system to identify and neutralize foreign substances. The presence of antibodies in semen is related to the breakdown of the blood–testis barrier, among other things [49].

Therefore, the high abundance of protein FAM3C, hemoglobin beta, and Ig lambda-1 chain C regions in the SP of bulls classified as UNFIT might be related to the establishment of an oxidative stress state capable of decreasing the parameters of sperm quality, as observed in this group of animals. We also highlight that both FAM3C and hemoglobin beta would have strong and positive correlations with major sperm defects.

**Figure 6:**
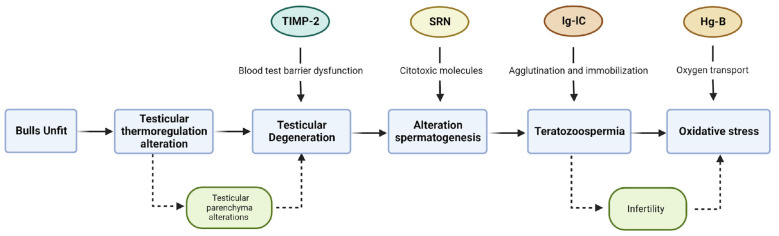
Functional role of the differentially abundant proteins (DAP) in sperm defect generation. Young Nellore bulls classified as UNFIT for reproduction showed nine up-regulated proteins in the seminal plasma. Among them, the proteins TIMP-2 (A0A0E3UT00), SRN (P00669), Ig-C (L8IAF4), and Hg-B (D4QBD1) participate in mechanisms that could result in sperm defects.

Finally, the profile of DAPs in the group of UNFIT bulls suggests cellular mechanisms in response to oxidative stress. The alterations in the testicular parenchyma might cause oxidative stress due to the initiation of a process of testicular degeneration (Figure 6). We theorized that these alterations might partly explain the higher percentage of sperm pathology counts observed in this group of bulls.

## 5. Conclusions

LC-MS/MS proteomics identified 297 proteins in the seminal plasma (SP) of young Nellore bulls classified as FIT and UNFIT for reproduction, among which 93.6% are proteins with an abundance lower than 1%, referred to here as low abundance. Our results showed a clear distinction between the evaluated FIT and UNFIT groups of young bulls, despite 11 of the most abundant proteins present in the seminal plasma of all FIT and UNFIT bulls. These 11 proteins were detected as differentially abundant proteins (DAPs), and were in accordance with the characteristics described for molecular markers for reproduction in males, as all of them are related to reproduction events, found in the early stage of evaluation (in young bulls), and can be easily detected, measured, evaluated, and compared. Spermadhesin-1 was the protein with the highest mean abundance in both groups, being a strong candidate as a molecular marker in young bulls approved for reproduction: FIT bulls. Quantitative proteomics analysis revealed that Spermadhesin-1 and Ig gamma-1 chain C region were up-regulated in the group of FIT bulls, while Spermadhesin Z13, metalloproteinase inhibitor 2, seminal plasma protein BSP-30 kDa, vasoactive intestinal peptide, hemoglobin beta, Spermadhesin 2, seminal ribonuclease, protein FAM3C, and Ig lambda-1 chain C regions were up-regulated in the group of UNFIT bulls. The up-regulated proteins in UNFIT bulls are associated with oxidative stress, which may be related to the higher percentage of sperm defects since oxidative stress can cause structural alterations in various stages of spermatogenesis, thus altering sperm morphology.

## Figures and Tables

**Table 1:** Semen phenotypic characteristics of Nellore bulls classified as FIT and UNFIT for reproduction. Physical and morphological parameters of the semen of Nellore bulls collected by the electroejaculation method and evaluated by microscopy.

Variable	FIT Bulls	UNFIT Bulls	Mean	CV	V. Min	V. Max
PE	34.91 ± 1.22	34.51 ± 1.52	34.61 ± 1.39	3.99	31	36.6
Vol	3.40 ± 0.69	4.70 ± 1.33	4.05 ± 1.23	26.35	2	6
Asp	2.70 ± 0.67	2.60 ± 0.51	2.65 ± 0.58	22.68	2	4
MM	1.65 ± 1.49	0.70 ± 0.82	1.17 ± 1.27	102.53	0	4
Mot	70.00 ± 11.05	61.00 ± 23.66	65.00 ± 18.56	28.2	0	90
Vig	3.15 ± 0.34	2.80 ± 1.01	2.97 ± 0.75	-	0	4
Morp	12.30 ± 3.27 b	32.90 ± 9.48 a	22.60 ± 12.62	31.37	9	50

CV: Coefficient of variation; PE: scrotal perimeter (cm); Vol: ejaculate volume (mL); Asp: Aspect; MM: mass motility (0–5); Mot: sperm motility (%); Vig: Vigor (0–5); Morp: Morphology (%). Mean values followed by different lowercase letters indicate difference (*p* < 0.05).

**Table 2:** The 19 most abundant proteins in the seminal plasma of Nellore bulls are classified as FIT and UNFIT for reproduction. Protein abundance for FIT bulls represents 70.5%, while for UNFIT, 72.1% of the total abundance of the 297 identified proteins. The other 278 proteins identified in our study were low-abundance proteins, both for bulls of the FIT and UNFIT groups.

UniProtKb ID	Protein	Avg. FIT	%	Avg. UNFIT	%
P29392	Spermadhesin-1	428.58	**47.7%**	240.36	**25.7%**
Q8HZY1	Serine protease inhibitor clade E member 2	34.75	**3.9%**	36.74	**3.9%**
P02784	Seminal plasma protein PDC-109	20.32	**2.3%**	76.44	**8.2%**
A0A4W2BP54	Jacalin-type lectin domain-containing protein	14.70	**1.6%**	22.09	**2.4%**
G3MWX7	Lipoclin_cytosolic_FA-bd_dom	13.69	**1.5%**	18.87	**2.0%**
P17697	Clusterin	13.23	**1.5%**	6.43	0.7%
P80311	Peptidyl-prolyl cis-trans isomerase B	12.12	**1.3%**	18.63	**2.0%**
A0A0E3UT00	Metalloproteinase inhibitor 2	11.79	**1.3%**	37.80	**4.0%**
L8HPF5	Ig gamma-1 chain C region	11.54	**1.3%**	3.31	0.4%
P81019	Seminal plasma protein BSP-30 kDa	11.05	**1.2%**	32.77	**3.5%**
P79345	NPC intracellular cholesterol transporter 2	10.87	**1.2%**	20.18	**2.2%**
F1MCF5	Glutathione peroxidase	10.69	**1.2%**	9.20	**1.0%**
D4QBC5	Hemoglobin beta	9.02	**1.0%**	4.86	0.5%
P82292	Spermadhesin Z13	7.80	0.9%	50.14	**5.4%**
F1MGQ1	Deoxyribonuclease	7.15	0.8%	17.13	**1.8%**
D4QBD1	Hemoglobin beta	6.93	0.8%	18.93	**2.0%**
W5P198	Vasoactive intestinal peptide	4.46	0.5%	28.53	**3.0%**
Q4R0H2	Spermadhesin 2	3.46	0.4%	21.71	**2.3%**
P00669	Seminal ribonuclease	1.95	0.2%	10.68	**1.1%**
	Other proteins		29.5%		27.9%

AVG: average of spectral counts.

**Table 3:** Principal annotations from the analysis of functional clusters of seminal plasma proteins from Nellore bulls. Clusters were based on gene ontology (GO) terms of proteins from SP bulls classified as FIT and UNFIT for reproduction associated with biological processes (BP), molecular function (MF), and cellular components (CC).

GoTerm	Functional Annotation Clusters	Number of Protein	Enrichment Score
BP	Phospholipid efflux	4	3.12
	Sperm capacitation	4	
	Positive regulation of sperm capacitation	3	
MF	ATP binding	16	4.42
	Unfolded protein binding	11	
	ATPase activity	10	
	Protein binding involved in protein folding	8	
CC	chaperonin-containing T-complex	4	2.04
	Cell body	4	
	Microtubule	4	

Enrichment Score: a metric that quantifies the over-representation or under-representation of specific functional categories or terms within a set of proteins compared to a reference or random distribution.

## Data Availability

The data presented in this study are available from the corresponding author upon request.

## Supplementary Materials

The following supporting information can be downloaded at: https://www.mdpi.com/article/10.3390/vetsci10100610/s1, Table S1. Label-free quantification data of the seminal plasma (SP) proteins of Nellore bulls classified as FIT and UNFIT for reproduction. Only the proteins identified in at least three replicates of each group were considered for quantitative analysis. The abundance of the proteins was estimated by the Exponentially Modified Protein Abundance Index (emPAI). Table S2. Classification of the SP proteins according to the functional categories of the KOG database. The list presents the identification code of the proteins in the UniProtKB database (UniProtKB ID), the identification code of the proteins in the KOG (KOG ID), the description of the protein in the KOG (KOG Description), the classification of the proteins into KOG groups, and the E-value of the analysis for the classification of the proteins in the different KOG groups.
