# Supplementary material for: Relative Abundance of Spermadhesin-1 in the Seminal Plasma of Young Nellore Bulls Is in Agreement with Reproductive Parameters
Source: Vet Sci. 2023 Oct 7;10(10):610. doi: 10.3390/vetsci10100610 (PMC10611397; doi:10.3390/vetsci10100610)
Supplement: Supplementary file 1 [file vetsci-10-00610-s001.zip › vetsci-2561924-supplementary.pdf]

Relative Abundance of Spermadhesin-1 in the Seminal Plasmaof Young Nellore Bulls Is in Agreement with Reproductive Parameters

Camilo José Ramírez-López 1,2,3,* , Edvaldo Barros 4 , Pedro Marcus Pereira Vidigal 4 , Denise Silva Okano 1, Juliana Nascimento Duarte Rodrigues 1, Lidiany Lopes Gomes 1, José Carlos Montes-Vergara 2, Victor Gerardo Petro Hernandez 5, Maria Cristina Baracat-Pereira 3 , Simone Eliza Facioni Guimarães 6 and José Domingos Guimarães 1

1 Laboratory of Animal Reproduction, Department of Veterinary Medicine, Universidade Federal de Viçosa,

Viçosa 36570-900, Brazil; deniseokano@gmail.com (D.S.O.); juliana.nascimento@ufv.br (J.N.D.R.);

lidianyveterinaria@gmail.com (L.L.G.); jdguima@ufv.br (J.D.G.)

2 Department of Animal Science, Universidad de Córdoba, Monteria 230002, Colombia;

josemontesv@correo.unicordoba.edu.co

3 Laboratory of Proteomics and Protein Biochemistry, Department of Biochemistry and Molecular Biology,

Universidade Federal de Viçosa, Viçosa 36570-900, Brazil; baracat@ufv.br

4 Nucleus for Analysis of Biomolecules, Universidade Federal de Viçosa, Viçosa 36570-900, Brazil;

edvaldo.barros@ufv.br (E.B.); pedro.vidigal@ufv.br (P.M.P.V.)

5 Department of Veterinary Medicine, Universidad de Santander, Valledupar 200001, Colombia;

vic.petro@mail.udes.edu.co

6 Laboratory of Animal Biotechnology, Department of Animal Science, Universidade Federal de Viçosa,

Viçosa 36570-900, Brazil; sfacioni@ufv.br

* Correspondence: camilo.lopez@ufv.br

Table S1. Label-free quantification data of the seminal plasma (SP) proteins of Nellore bulls classified as FIT and UNFIT for reproduction. Only the proteins identified in at least three replicates of each group were considered for quantitative analysis. The abundance of the proteins was estimated by the Exponentially Modified Protein Abundance Index (emPAI).

UniProtKB.ID	Description	Spectral count					
		FIT.R1	FIT.R2	FIT.R3	UNFIT.R1	UNFIT.R2	UNFIT.R3
A0A0A0MP92	Serpin A3-7	0.40215	0.25027	0.16057	0.45102	0.34691	0.14477
A0A0A7NM57	Preprocathelicidin antimicrobial peptide	10998.0	17612.0	0.50121	0.83934	0.83934	0.20377
A0A0E3UT00	Metalloproteinase inhibitor 2	14067.0	13348.0	79672.0	25853.0	57779.0	29765.0
A0A0M4B6N2	Binder of sperm 3	21093.0	24637.0	24637.0	24637.0	24637.0	15737.0
A0A4W2BP54	Jacalin-type lectin domain-containing protein	14072.0	15021.0	15021.0	22812.0	18532.0	24931.0
A0A4W2DWN4	Glutathione peroxidase	2472.0	29246.0	34996.0	57814.0	49148.0	13901.0
A0A6P5C9R4	protein LEG1 homolog	0.99688	0.76962	0.76962	0.60904	0.76962	0.54071
A0JN47	Plexin domain containing 2	0.11329	0.12556	0.060924	0.19413	0.060924	0.17467
A0JNP2	Secretoglobin family 1D member	12117.0	13779.0	0.78154	13779.0	13779.0	0.69753
A2I7M9	Serpin A3-2	0.51029	0.45994	0.57471	0.45994	0.16341	0.22894
A2I7N1	Serpin A3-5	0.40772	0.35161	0.45735	0.25354	0.078234	0.22775
A3KMY8	Beta-glucuronidase	0.29418	0.20872	0.099419	0.15278	0.048532	0.35102
A4IFC0	Platelet-derived growth factor D	12841.0	16968.0	16968.0	16968.0	0.78373	16543.0
A4IFR2	PCSK1N protein	0.41063	0.28666	0.13431	0.0	0.0	0.12151
A5D7U1	Placenta-expressed transcript 1 protein	0.42242	0.4728	0.67569	1468.0	0.90652	10233.0
A5D984	Pyruvate kinase	0.17931	0.27423	0.27423	0.062459	0.0	0.11623
A5PJL8	C1QTNF5 protein	13725.0	15832.0	15832.0	15832.0	12557.0	13725.0
A5PKI3	Protein FAM3C	0.28683	0.51509	0.31914	42693.0	59509.0	48431.0
A6BMK7	Sialidase-1	0.32244	0.25965	0.079982	0.25965	0.16636	0.2332
A6QLR7	ARSG protein	0.057863	0.063947	0.0	0.0	0.0	0.0
A6QLS9	RAB10 protein	0.14801	0.35398	0.0	0.35398	0.35398	0.31793
A6QLZ3	ARSB protein	0.23992	0.061039	0.061039	0.1258	0.0	0.11352
A6QM01	N-acetyl-alpha-glucosaminidase	0.53988	0.68811	0.099881	0.26872	0.048752	0.18849
A6QNJ8	GANAB protein	0.96805	0.7943	0.29671	0.52535	0.17632	0.4662
A6QNL5	Protein disulfide-isomerase A6	0.2118	0.073087	0.15152	0.073087	0.15152	0.066132
A6QNX2	DPP7 protein	0.42769	0.29892	0.29892	0.48039	0.21671	0.26791
A6QP36	LMAN2 protein	0.081467	0.090039	0.0	0.18819	0.090039	0.16957
A6QP39	MSLN protein	11978.0	22622.0	10329.0	10329.0	1.38	13609.0
A6QPK0	SCGB2A2 protein	63679.0	54643.0	15425.0	78229.0	78229.0	21306.0

A6QPT4	MPO protein	0.42196	0.18833	0.044082	0.0	0.0	0.03989
A6QPZ4	SERPINB4 protein	12668.0	19472.0	12494.0	0.56888	10556.0	0.50559
A6QQA8	Sulfhydryl oxidase	82976.0	73527.0	56802.0	94439.0	83399.0	51988.0
A7E3W2	Galectin-3-binding protein	12756.0	1772.0	13388.0	14751.0	0.97333	1795.0
A7MB70	Semaphorin-3C	0.57022	0.33683	0.18044	0.45243	0.28252	0.3011
A7MBJ5	Cullin-associated NEDD8-dissociated protein 1	0.098906	0.053423	0.053423	0.0	0.0	0.073298
A8DC37	Fc fragment of IgG receptor IIa	0.22251	0.3929	0.24723	0.1168	0.1168	0.10567
B0JYQ0	ALB protein	31759.0	49246.0	35949.0	49246.0	35949.0	31759.0
B5T255	Peptidoglycan recognition protein 1	27576.0	16337.0	2637.0	59362.0	3274.0	1.8
B9TUD2	Cathelicidin 7	0.63362	0.71279	0.0	0.0	0.71279	0.0
D4QBC5	Hemoglobin beta	89607.0	9047.0	9047.0	43549.0	56046.0	46068.0
D4QBD1	Hemoglobin beta	46068.0	71459.0	9047.0	17.85	22249.0	16695.0
E1B6Z6	Lipocalin 2	23845.0	11035.0	0.81285	14409.0	11035.0	12542.0
E1B748	Hypoxia up-regulated 1	0.8819	0.76968	0.88556	0.8267	0.55886	0.53895
E1B818	Olfactomedin like 2A	0.35526	0.27051	0.27051	0.33283	0.15448	0.24252
E1B8A0	Tartrate-resistant acid phosphatase type 5	0.39776	0.20234	0.096511	0.31838	0.096511	0.0
E1B9P4	Epididymal sperm binding protein 1	0.0	0.0	0.0	0.30274	0.30274	0.0
E1BBK6	Nephronectin	0.34223	0.54126	0.17613	0.54126	0.24148	0.2168
E1BBU4	ADAM metallopeptidase domain 15	0.071234	0.078859	0.038682	0.038682	0.038682	0.035004
E1BDF3	Matrilin 4	34336.0	23328.0	19873.0	21553.0	16776.0	24591.0
E1BDR2	Alpha-L-iduronidase	0.14348	0.21787	0.10357	0.27939	0.0	0.19575
E1BDY3	Golgi apparatus protein 1	0.10027	0.1409	0.082302	0.11121	0.026714	0.10027
E1BG25	Melanotransferrin	0.37325	0.35807	0.41877	0.35807	0.091384	0.21925
E1BH06	Complement component 4A	0.4204	0.44626	0.27105	0.13785	0.096635	0.18189
E1BHP0	Alpha-mannosidase	0.35902	0.36032	0.20277	0.16633	0.0	0.087262
E1BI28	GM2 ganglioside activator	0.34605	0.91961	0.17707	0.0	16596.0	0.56168
E1BI74	NAD	0.9265	0.85567	10571.0	15279.0	10571.0	18022.0
E1BKX1	Hyaluronoglucosaminidase	0.043046	0.097504	0.047618	0.023532	0.0	0.0
E1BKZ9	Sortilin 1	0.072294	0.12242	0.039247	0.039247	0.039247	0.11038
E1BLI4	CUB domain-containing protein	22817.0	46661.0	46661.0	46661.0	35617.0	30006.0
E1BMJ0	Serpin family G member 1	0.96635	0.60649	0.50129	0.96838	0.22527	0.6352
E1BN79	Carboxylic ester hydrolase	13254.0	14001.0	12723.0	15351.0	0.72838	0.81428
E1BNJ9	Serine protease 22	0.31976	0.22566	0.35692	0.35692	0.50224	0.096895
E1BQ21	FAM3 metabolism regulating signaling molecule B	10589.0	0.48692	0.69714	15235.0	22875.0	16193.0
F1MCF5	Glutathione peroxidase	9432.0	15137.0	74937.0	12029.0	95197.0	60573.0
F1MD95	Calsyntenin 1	0.030628	0.068834	0.033844	0.033844	0.033844	0.094726

F1MDF2	Beta-microseminoprotein	0.63102	19116.0	19116.0	1229.0	19116.0	16602.0
F1MGN0	Semaphorin 3F	0.15478	0.21947	0.12642	0.26883	0.0826	0.074606
F1MGQ1	Deoxyribonuclease	62293.0	10.86	43749.0	13455.0	22704.0	15238.0
F1MGU7	Fibrinogen gamma-B chain	0.3726	0.62975	0.41748	0.14976	0.14976	0.20928
F1MHF1	ST6 beta-galactoside alpha-2,6-sialyltransferase 1	11257.0	11276.0	0.97291	11276.0	12945.0	17964.0
F1MHR8	MATN2 protein	0.061929	0.033703	0.033703	0.068541	0.0	0.094317
F1MHS5	Protein S100-A9	0.20665	0.50902	0.85372	0.0	0.0	0.20665
F1MLR4	Ciliary neurotrophic factor receptor	0.081014	0.089539	0.1871	0.29339	0.1871	0.16859
F1MLW2	BPI fold-containing family B member 1	28665.0	48168.0	21622.0	26208.0	26208.0	42578.0
F1MLW8	Immunoglobulin lambda-like polypeptide 5	0.46216	0.51781	0.32072	0.32072	0.74431	0.28824
F1MNJ4	APC down-regulated 1	0.25619	0.28573	0.20743	0.28573	0.1339	0.1208
F1MS23	Lipocln_cytosolic_FA-bd_dom domain-containing protein	0.37003	0.4124	0.4124	0.99486	29795.0	0.60359
F1MTI7	Protein CutA	0.45251	0.84647	0.84647	0.50509	12653.0	0.75057
F1MU34	Aminopeptidase	0.094114	0.068396	0.033632	0.033632	0.0	0.12741
F1MUB9	Serine protease 8	0.29509	0.20888	0.20888	0.32915	0.20888	0.29509
F1MUF4	Beta-galactosidase	0.68525	12597.0	0.69471	0.8653	0.61536	0.68525
F1MWD3	T-complex protein 1 subunit epsilon	0.11286	0.26579	0.060694	0.060694	0.0	0.05492
F1MWI1	Clusterin	12697.0	19053.0	12714.0	10925.0	0.9277	0.95547
F1MWT0	Alpha-mannosidase	0.31954	0.20151	0.1653	0.063104	0.0	0.057024
F1MX50	Cellular repressor of E1A stimulateds 1	0.4771	0.77032	0.33053	1042.0	1042.0	0.9158
F1MY12	NTR domain-containing protein	0.46704	0.52334	0.52334	0.32393	0.52334	0.46704
F1MYX5	Lymphocyte cytosolic protein 1	0.37605	0.49513	0.051562	0.1628	0.0	0.31471
F1MZ40	Growth arrest specific 6	0.40892	0.32778	0.15229	0.099113	0.048386	0.0
F1MZJ5	ADAM metallopeptidase domain 9	0.0	0.12776	0.0	0.0	0.0	0.075377
F1N0F2	Sialic acid acetylesterase	0.37741	0.42318	0.12483	0.19297	0.19297	0.30583
F1N152	Serine protease HTRA1	0.63351	0.50001	0.40199	0.50001	0.22475	0.73685
F1N303	ADAM metallopeptidase with thrombospondin type 1 motif 19	0.049266	0.0	0.026892	0.0	0.0	0.074802
F1N3U5	Vanin 2	14763.0	19026.0	21025.0	42858.0	36266.0	21537.0
F1N554	Polypeptide N-acetylgalactosaminyltransferase	0.051614	0.11733	0.05704	0.11733	0.05704	0.10589
F1N5H2	Alpha-L-fucosidase	0.26791	0.21671	0.067569	0.21671	0.29892	0.19486
F1N5W4	Ectonucleotide pyrophosphatase	0.2556	0.18183	0.0	0.087118	0.087118	0.078825
F1N7D7	Dystroglycan 1	0.4371	0.43859	0.24383	0.43859	0.33767	0.3454
F1N7F3	Receptor protein-tyrosine kinase	0.024729	0.027326	0.027326	0.027326	0.0	0.0
G1CW25	Lactotransferrin	37363.0	45569.0	38532.0	32387.0	18237.0	2705.0
G3MWX7	Lipocln_cytosolic_FA-bd_dom domain-containing protein	15185.0	11027.0	14855.0	26555.0	14855.0	15185.0
G3MX66	Vitelline membrane outer layer 1 homolog	13958.0	0.89492	12233.0	0.89492	0.61508	0.33808

G3MZ03	Alpha 1,4-galactosyltransferase	0.35565	0.087354	0.18234	0.52004	0.087354	0.25635
G3N0V0	Immunoglobulin heavy constant mu	18662.0	18924.0	13845.0	16262.0	13845.0	14049.0
G3N2D8	Glutathione hydrolase	0.0	0.12246	0.059462	0.059462	0.1892	0.11051
G3X6G7	Meteorin-like protein	0.097543	0.22724	0.10781	0.10781	0.0	0.097543
G5E589	Proteasome subunit beta	0.42856	0.47976	0.13954	0.13954	0.47976	0.60891
G5E5M8	Alpha-mannosidase	0.18214	0.16633	0.13098	0.03125	0.03125	0.05736
G5E5T5	Immunoglobulin heavy constant mu	0.25122	0.50871	0.1788	0.27986	0.50871	0.34827
G5E604	Ig-like domain-containing protein	40373.0	33495.0	22415.0	33495.0	33495.0	28474.0
H7BWW2	Beta-hexosaminidase	11826.0	0.99012	1232.0	0.58217	0.8792	0.96685
L8HKR7	Ig gamma-3 chain C region	0.76386	0.68236	10715.0	0.86682	12986.0	0.45985
L8HP94	Ig alpha-1 chain C region	0.82498	13423.0	13423.0	11309.0	0.7637	13617.0
L8HPF5	Ig gamma-1 chain C region	13096.0	90735.0	12446.0	46544.0	13779.0	38916.0
L8HZ66	Phosphoglycerate kinase	1896.0	15517.0	0.7271	10189.0	0.7271	0.64251
L8HZP9	Angiotensin-converting enzyme	0.43909	0.42124	0.386	0.35163	0.25356	0.19962
L8I064	Iduronate 2-sulfatase	0.24597	0.27423	0.0	0.12882	0.062459	0.11623
L8I2G8	SERPIN domain-containing protein	0.0	0.0	0.0	0.0	0.10745	0.097218
L8IAF4	Ig lambda-1 chain C regions	12278.0	22072.0	0.33823	47437.0	92862.0	54822.0
L8IAH9	Sulfhydryl oxidase	61842.0	49755.0	41692.0	59075.0	55817.0	38429.0
L8IC40	Protein FAM3D	0.9158	0.77032	0.53475	0.77032	13555.0	14848.0
L8ICQ2	5'-nucleotidase	33925.0	22583.0	24468.0	38304.0	41099.0	25815.0
L8IEA1	Beta-N-acetylhexosaminidase	15194.0	0.84186	0.84186	0.72101	0.72101	0.6369
L8IHR1	Protocadherin Fat 2	0.013572	0.015001	0.022586	0.0	0.0	0.0
L8IQM7	Vitamin K-dependent protein S	0.29078	0.26433	0.45537	0.048025	0.0	0.13612
L8IVU7	T-complex protein 1 subunit delta	0.54481	0.27079	0.34925	0.19689	0.0	0.31234
L8IXW5	Proactivator polypeptide	0.9154	0.7112	0.92817	0.92817	10468.0	0.81442
L8J2B5	Amyloid-like protein 2	0.076665	0.041578	0.041578	0.041578	0.084884	0.037625
L8J3B9	Slit-like protein 3 protein	0.33724	0.28664	0.31646	0.071154	0.0	0.0
L8J3G6	Annexin	0.76486	10433.0	0.86873	0.42945	0.86873	0.91406
O46375	Transthyretin	11737.0	0.23679	0.52965	0.52965	0.89186	0.21422
O77588	Procollagen-lysine,2-oxoglutarate 5-dioxygenase 1	0.84687	0.73281	0.73281	0.96719	0.40257	0.41212
P00517	cAMP-dependent protein kinase catalytic subunit alpha	0.08405	0.092895	0.092895	0.092895	0.092895	0.17516
P00570	Adenylate kinase isoenzyme 1	1049.0	0.87724	0.87724	0.87724	1572.0	0.77513
P00669	Seminal ribonuclease	0.20665	32212.0	24363.0	10808.0	16819.0	4423.0
P01045	Kininogen-2	0.097406	0.0	0.10791	0.10791	0.0	0.14961
P01888	Beta-2-microglobulin	4.36	74662.0	35987.0	74662.0	52397.0	20627.0
P01966	Hemoglobin subunit alpha	23359.0	14087.0	14087.0	80044.0	36571.0	30777.0

P02584	Profilin-1	0.49836	0.55669	0.94225	0.0	0.94225	0.49836
P02672	Fibrinogen alpha chain	0.94971	0.60508	0.37087	0.60508	0.69172	0.26929
P02676	Fibrinogen beta chain	0.57106	0.28262	0.28262	0.20524	0.28262	0.1846
P02769	Albumin	37953.0	43519.0	35949.0	55586.0	43519.0	33729.0
P02784	Seminal plasma protein PDC-109	22817.0	19809.0	38878.0	93923.0	116.9	18.51
P04557	Seminal plasma protein A3	15896.0	55244.0	33006.0	42971.0	55244.0	45441.0
P05307	Protein disulfide-isomerase	0.32124	0.44538	0.20224	0.20224	0.063319	0.18192
P07456	Insulin-like growth factor II	0.17048	0.18844	0.0	0.0	0.0	0.0
P07688	Cathepsin B	0.9928	15818.0	15818.0	15818.0	13481.0	18132.0
P08037	Beta-1,4-galactosyltransferase 1	0.0	0.0	0.081191	0.0	0.0	0.15232
P0C0S9	Histone H2A type 1	0.2387	0.59729	0.59729	0.26384	0.0	0.0
P10096	Glyceraldehyde-3-phosphate dehydrogenase	0.19247	0.21372	0.0	0.10169	0.21372	0.19247
P10152	Angiogenin-1	0.67684	11284.0	27505.0	44716.0	44716.0	0.67684
P12763	Alpha-2-HS-glycoprotein	0.38985	0.31224	0.0	0.1986	0.0	0.0
P13600	Beta-nerve growth factor	30936.0	31355.0	37052.0	26348.0	59299.0	22366.0
P13696	Phosphatidylethanolamine-binding protein 1	0.34402	0.38322	0.0	0.0	0.62682	0.0
P14769	N-acetyllactosaminide alpha-1,3-galactosyltransferase	0.1581	0.08416	0.08416	0.27433	0.08416	0.076149
P15497	Apolipoprotein A-I	10661.0	14889.0	0.40769	0.25604	0.57764	0.23039
P16116	Aldo-keto reductase family 1 member B1	0.55282	0.47311	0.33714	0.62291	0.33714	0.55282
P17697	Clusterin	12665.0	13511.0	13511.0	7978.0	58239.0	54738.0
P19120	Heat shock cognate 71 kDa protein	0.3693	0.34584	0.28084	0.34584	0.34584	0.30918
P19879	Mimecan	17569.0	12509.0	0.66046	0.66046	10338.0	0.90662
P22226	Cathelicidin-1	47182.0	3612.0	15997.0	21472.0	21472.0	0.68724
P25326	Cathepsin S	11107.0	1493.0	0.72996	10767.0	10767.0	0.64545
P28291	C-C motif chemokine 2	28474.0	48361.0	22415.0	95075.0	68309.0	28474.0
P29392	Spermadhesin-1	365.61	408.91	511.22	261.52	261.52	198.04
P31096	Osteopontin	31619.0	43656.0	24281.0	24281.0	20649.0	17692.0
P31976	Ezrin	0.26253	0.16672	0.22826	0.16672	0.10827	0.20502
P34955	Alpha-1-antiproteinase	0.85877	0.83465	0.5764	0.35449	0.5764	0.85877
P37141	Glutathione peroxidase 3	32883.0	30372.0	30372.0	79621.0	39279.0	41442.0
P38657	Protein disulfide-isomerase A3	0.25071	0.53943	0.20309	0.36091	0.44742	0.65425
P40682	V-type proton ATPase subunit S1	0.53771	0.8395	0.22527	0.60649	0.71905	0.44603
P45478	Palmitoyl-protein thioesterase 1	0.23315	0.77896	0.41286	0.25912	0.1221	0.23315
P52193	Calreticulin	0.21921	0.075485	0.075485	0.075485	0.0	0.0683
P55206	C-type natriuretic peptide	36269.0	43252.0	43252.0	43252.0	43252.0	36269.0
P62803	Histone H4	11806.0	31299.0	2.11	0.76351	2.11	0.29676

P62935	Peptidyl-prolyl cis-trans isomerase A	0.41144	0.4589	0.4589	0.20785	0.4589	0.0
P62992	Ubiquitin-40S ribosomal protein S27a	0.18684	0.75632	0.75632	1119.0	0.45569	0.67175
P63103	14-3-3 protein zeta/delta	0.57084	0.451	0.64269	0.0	0.28167	0.11952
P63258	Actin, cytoplasmic 2	51776.0	31372.0	25007.0	17247.0	15064.0	14855.0
P68103	Elongation factor 1-alpha 1	0.20928	0.14976	0.072267	0.0	0.072267	0.13506
P79345	NPC intracellular cholesterol transporter 2	5242.0	80853.0	19269.0	19269.0	29274.0	11985.0
P80025	Lactoperoxidase	0.082523	0.044693	0.091384	0.091384	0.14016	0.040444
P80311	Peptidyl-prolyl cis-trans isomerase B	71594.0	16838.0	12373.0	16838.0	30741.0	83033.0
P81019	Seminal plasma protein BSP-30 kDa	67593.0	14334.0	12059.0	46.19	39189.0	12933.0
P81134	Renin receptor	18388.0	24446.0	16426.0	0.85595	12146.0	0.61859
P81187	Complement factor B	1858.0	21842.0	24589.0	12871.0	0.93834	11172.0
P81265	Polymeric immunoglobulin receptor	0.364	0.34926	0.1867	0.9001	0.53409	0.72157
P81425	Dipeptidyl peptidase 4	0.03691	0.040788	0.08324	0.22127	0.12742	0.15602
P81947	Tubulin alpha-1B chain	0.28694	0.071945	0.071945	0.0	0.0	0.065098
P82292	Spermadhesin Z13	87206.0	63076.0	83702.0	86801.0	52402.0	11203.0
Q08DW4	Mannan-binding lectin serine peptidase 1	0.21762	0.044385	0.044385	0.090741	0.044385	0.040165
Q08E54	Amyloid-beta A4 protein	0.21633	0.13836	0.044142	0.13836	0.044142	0.21633
Q0IIH5	Nucleobindin 2	0.47348	0.64566	0.0	0.53262	0.3293	0.57182
Q0P569	Nucleobindin-1	75278.0	42569.0	36269.0	33408.0	36269.0	32554.0
Q0V8B6	Tripeptidyl-peptidase 1	0.68259	0.77446	0.67556	0.58217	0.33209	0.59728
Q0V8R6	Beta-hexosaminidase subunit alpha	0.29775	0.49506	0.33277	0.33277	0.33277	0.36719
Q0VCQ9	Reticulocalbin 2	0.089464	0.32694	0.32694	0.09888	0.09888	0.089464
Q0VCX2	Endoplasmic reticulum chaperone BiP	21517.0	25455.0	16475.0	25455.0	12877.0	21517.0
Q0VD19	Sphingomyelin phosphodiesterase	0.24744	0.21533	0.21533	0.1575	0.10242	0.14186
Q148N3	Matrix metallopeptidase 7	0.23597	0.41817	0.41817	0.12351	0.26227	0.23597
Q17QB3	Acid ceramidase	0.70678	0.67401	0.67401	0.67401	0.93949	0.49326
Q17QC7	Nectin cell adhesion molecule 2	0.49407	0.46114	0.20877	0.37165	0.20877	0.33214
Q17QK3	Carboxypeptidase Q	0.17837	0.198	0.198	0.0	0.09453	0.17837
Q17QR9	SPARC	0.25266	0.1798	0.28148	0.086186	0.0	0.16204
Q1JPB0	Leukocyte elastase inhibitor	0.96563	0.93747	0.51189	0.1798	0.1798	0.16204
Q1LZH9	N-acetylglucosamine-6-sulfatase	0.66285	0.6561	0.56583	0.48048	0.11863	0.28951
Q1RMN0	Aspartylglucosaminidase	0.29118	0.32476	0.32476	0.75498	0.59794	0.29118
Q1RMN8	Immunoglobulin light chain, lambda gene cluster	0.66323	13121.0	0.74852	0.74852	10107.0	0.66323
Q1ZYR0	Ceroid-lipofuscinosis neuronal protein 5	0.25861	0.28816	0.088071	0.088071	0.088071	0.079686
Q28017	Platelet-activating factor acetylhydrolase	76158.0	76975.0	90001.0	11329.0	55794.0	41907.0
Q28085	Complement factor H	0.98843	0.88169	12461.0	19664.0	0.92988	0.85635

Q29443	Serotransferrin	15771.0	15938.0	17143.0	19722.0	14787.0	14732.0
Q29444	Beta-mannosidase	15108.0	19614.0	16661.0	14002.0	10146.0	14324.0
Q29451	Lysosomal alpha-mannosidase	0.12045	0.1337	0.098685	0.064753	0.031869	0.02884
Q2HJF0	Serotransferrin-like	16374.0	17683.0	10396.0	12583.0	10396.0	0.90889
Q2KHZ8	Lysosomal acid glucosylceramidase	0.054714	0.2647	0.12459	0.060466	0.0	0.11242
Q2KIF2	Leucine-rich alpha-2-glycoprotein 1	0.38985	0.1986	0.0	0.0	0.0	0.0
Q2KIM0	Tissue alpha-L-fucosidase	0.26251	0.21244	0.29284	0.13703	0.0	0.33827
Q2KIY5	Putative phospholipase B-like 2	0.21441	0.2388	0.11301	0.11301	0.11301	0.15684
Q2KJF1	Alpha-1B-glycoprotein	0.60916	0.80336	0.48157	0.58189	0.38761	0.42873
Q2LGB2	MD-2 protein	0.38455	0.4287	0.4287	0.19528	0.19528	0.91697
Q2NKZ9	Carboxypeptidase	0.20632	0.31722	0.071308	0.071308	0.0	0.0
Q2NL29	Inositol-3-phosphate synthase 1	0.053905	0.18957	0.059572	0.0	0.0	0.053905
Q2T9S4	Glycerol-3-phosphate phosphatase	0.44426	0.10639	0.10639	0.0	0.22409	0.096255
Q2TBU0	Haptoglobin	0.64054	0.72482	0.86451	0.72482	0.47606	0.32693
Q2TBX4	Heat shock 70 kDa protein 13	15096.0	15757.0	0.60489	14074.0	1.25	0.96355
Q2UVX4	Complement C3	2572.0	33994.0	30776.0	22465.0	13064.0	13637.0
Q32KV0	Phosphoglycerate mutase 2	0.54966	13224.0	0.43493	0.43493	0.2722	0.54966
Q32L40	T-complex protein 1 subunit alpha	0.23605	0.12387	0.0	0.0	0.0	0.054408
Q32L48	Histone H2B type 1-N	0.54925	0.61406	0.61406	0.61406	10506.0	0.54925
Q32LF7	Beta-1,4-galactosyltransferase	92525.0	7333.0	66284.0	48524.0	7333.0	58636.0
Q32LQ0	Glutamyl aminopeptidase	0.0	0.032777	0.0	0.2135	0.032777	0.22705
Q3B7N2	Alpha-actinin-1	0.098125	0.10879	0.035022	0.0	0.0	0.064391
Q3MHM5	Tubulin beta-4B chain	0.37545	0.42071	0.23454	0.15081	0.072757	0.136
Q3MHN5	Vitamin D-binding protein	0.12684	0.14062	0.067997	0.0	0.0	0.0
Q3MHX6	Protein OS-9	0.13486	0.20443	0.1497	0.26176	0.097465	0.13486
Q3MI05	Lysosomal protective protein	0.60298	0.38395	0.21527	0.47687	0.1388	0.26609
Q3SX14	Gelsolin	0.42879	0.48217	0.29997	0.84435	0.29997	0.37325
Q3SX46	C1GALT1-specific chaperone 1	0.090576	0.21024	0.10011	0.10011	0.10011	0.29708
Q3SYR8	Immunoglobulin J chain	0.99215	11284.0	0.76213	0.76213	11284.0	0.41144
Q3SYW2	Complement C2	0.21318	0.13641	0.13641	0.088987	0.088987	0.080363
Q3SZB7	Fructose-1,6-bisphosphatase 1	0.41023	0.60455	0.32804	0.20821	0.099185	0.2941
Q3SZV7	Hemopexin	0.20066	0.2232	0.2232	0.30817	0.2232	0.20066
Q3T010	Phosphatidylethanolamine-binding protein 4	0.4527	0.3145	0.14652	0.7279	0.50709	0.64526
Q3T052	Inter-alpha-trypsin inhibitor heavy chain H4	0.099463	0.07223	0.035485	0.11028	0.0	0.032112
Q3T0A3	Complement factor D	0.40685	0.28415	0.64903	0.4552	0.64903	0.40685
Q3T0K2	T-complex protein 1 subunit gamma	0.22215	0.18039	0.0	0.056839	0.0	0.051432

Q3T0Z0	WAP four-disulfide core domain 2	0.60531	26436.0	26436.0	26436.0	26436.0	22651.0
Q3T149	Heat shock protein beta-1	0.74152	0.57889	0.35592	0.16444	0.35592	0.14877
Q3ZBD7	Glucose-6-phosphate isomerase	13154.0	10294.0	0.63227	0.31286	0.46388	0.413
Q3ZBH0	T-complex protein 1 subunit beta	0.11787	0.13065	0.20224	0.063319	0.0	0.057295
Q3ZBM0	A proliferation-inducing ligand	0.11952	0.13211	0.13211	0.28167	0.28167	0.40313
Q3ZBS7	Vitronectin	0.42769	0.21671	0.1397	0.21671	0.21671	0.12602
Q3ZBZ1	45 kDa calcium-binding protein	4426.0	49132.0	32151.0	1331.0	15368.0	13294.0
Q3ZC64	Ephrin-A1	15927.0	1846.0	1846.0	1451.0	1846.0	19707.0
Q3ZCH5	Zinc-alpha-2-glycoprotein	15364.0	24165.0	1513.0	53151.0	47005.0	26804.0
Q3ZCI9	T-complex protein 1 subunit theta	0.37822	0.60222	0.4241	0.26579	0.0	0.05492
Q4R0H2	Spermadhesin 2	39845.0	27383.0	36571.0	26.02	32661.0	64477.0
Q4W1E4	Angiotensin-converting enzyme	0.53152	0.67003	0.34872	0.53322	0.23823	0.26176
Q58CQ9	Pantetheinase	0.64635	0.62965	0.35694	0.35694	0.35694	0.39427
Q58DP6	Ribonuclease 4	25748.0	23075.0	23075.0	39279.0	12353.0	1.98
Q5DPW9	Cystatin E/M	20867.0	32212.0	17973.0	32212.0	41854.0	27246.0
Q5E946	Parkinson disease protein 7 homolog	0.16755	0.0	0.1852	0.1852	0.66485	0.36317
Q5E998	Cathepsin L2	35925.0	54495.0	30471.0	38764.0	2687.0	22729.0
Q5E9B1	L-lactate dehydrogenase B chain	0.41023	0.099185	0.0	0.20821	0.0	0.08974
Q5E9I4	Alpha-1,3-mannosyl-glycoprotein	0.20298	0.2258	0.07022	0.0	0.0	0.20298
Q5EA54	Solute carrier family 3	0.67568	0.65801	0.65801	0.76618	0.37165	0.77461
Q5EA62	Fibulin-5	0.3726	0.23285	0.23285	0.23285	0.14976	0.20928
Q5W5U3	Hexokinase	0.031866	0.071667	0.0	0.0	0.0	0.064748
Q6R8F2	Cadherin-1	0.29946	0.38416	0.24201	0.24201	0.15545	0.2576
Q6T182	Sex hormone-binding globulin	0.24559	0.38046	0.17493	0.083941	0.083941	0.15767
Q76LV2	Heat shock protein HSP 90-alpha	0.51552	0.7193	0.51719	0.28417	0.13321	0.12007
Q7SIH1	Alpha-2-macroglobulin	0.44054	0.40398	0.26273	0.1849	0.0	0.019397
Q7YRE5	GDP-fucose protein O-fucosyltransferase 2	0.12795	0.068576	0.068576	0.0	0.0	0.0
Q7YRQ8	Tissue factor pathway inhibitor 2	0.42242	0.90652	0.67569	0.4728	11692.0	0.79905
Q863C3	Gastrin-releasing peptide	0.84981	14534.0	0.9603	0.0	0.0	0.0
Q863K8	Galactosylgalactosylxylosylprotein	0.18574	0.32476	0.098277	0.20621	0.32476	0.088919
Q8HZY1	Serine protease inhibitor clade E member 2	43308.0	39897.0	21033.0	43184.0	36854.0	30191.0
Q8SPU5	BPI fold-containing family A member 1	0.60581	0.47742	0.68269	0.29719	0.68269	0.42649
Q95114	Lactadherin	0.39354	0.24521	0.24521	0.24521	0.24521	0.30406
Q95121	Pigment epithelium-derived factor	0.40999	0.16341	0.078616	0.078616	0.078616	0.14733
Q95M12	Legumain	0.47348	0.89737	0.3293	0.42735	0.76704	0.90798
Q95M18	Endoplasmin	0.63387	0.59071	0.26123	0.53035	0.26123	0.47071

Q9BEG4	Putative alpha-2,3-sialyltransferase	0.91406	0.56301	0.42945	0.42945	0.3073	0.50045
Q9BGI1	Peroxiredoxin-5, mitochondrial	0.49579	10893.0	0.55596	0.55596	0.55596	0.30792
Q9BGI2	Peroxiredoxin-4	0.50509	0.56763	0.40098	0.40098	0.7541	12653.0
Q9BGU5	Cathepsin D	51196.0	47264.0	42698.0	34628.0	17107.0	2608.0
Q9GLE5	72 kDa type IV collagenase	0.090458	0.10019	0.0	0.0	0.0489	0.090458
Q9MYM4	Lysosomal alpha-glucosidase	0.31758	0.22489	0.26701	0.31058	0.069955	0.16558
Q9N2I2	Plasma serine protease inhibitor	56262.0	64286.0	48941.0	64286.0	58772.0	5178.0
Q9XSC9	Transcobalamin-2	13652.0	17763.0	0.92789	19863.0	24553.0	17001.0
Q9XSJ4	Alpha-enolase	0.49602	0.67797	0.44731	0.076743	0.44731	0.22312
U5Y6U2	Osteopontin	46494.0	65083.0	32888.0	32888.0	24281.0	20661.0
W0UVF3	Ribonuclease A M1	18468.0	15997.0	0.46546	15997.0	15997.0	18468.0
W5NTH3	Adhesion G protein-coupled receptor G6	0.047193	0.052211	0.052211	0.052211	0.0	0.023324
W5NXW9	Immunoglobulin heavy constant mu	0.37545	0.63497	0.15081	0.42071	0.42071	0.21079
W5P198	Vasoactive intestinal peptide	85112.0	24383.0	24383.0	33074.0	19071.0	33453.0
W5P1A8	Proto-oncogene tyrosine-protein kinase receptor Ret	0.080148	0.12012	0.028766	0.0	0.0	0.026032
W5Q3V3	ATPase H+ transporting V1 subunit E2	0.27091	0.30133	0.0	0.0	0.14076	0.12735

Table S2. Classification of the SP proteins according to the functional categories of the KOG database. The list presents the identification code of the proteins in the UniProtKB database (UniProtKB ID), the identification code of the proteins in the KOG (KOG ID), the description of the protein in the KOG (KOG Description), the classification of the proteins into KOG groups, and the E-value of the analysis for the classification of the proteins in the different KOG groups.

UniProtKB ID	KOG ID	KOG Description	KOG Group	E-value
A0A0A0MP92	KOG2392	Serpin	Defense mechanisms	6.58E-125
A0A0A7NM57	---	no hits	no hits	---
A0A0E3UT00	KOG4745	Metalloproteinase inhibitor TIMP and related proteins	General function prediction only	4.03E-76
A0A0M4B6N2	---	no hits	no hits	---
A0A4W2DWN4	---	no hits	no hits	---
A0A6P5C9R4	---	no hits	no hits	---
A0JN47	KOG3848	Extracellular protein TEM7, contains PSI domain (tumor endothelial marker in humans)	Extracellular structures	0
A0JNP2	---	no hits	no hits	---
A2I7M9	KOG2392	Serpin	Defense mechanisms	4.46E-128
A2I7N1	KOG2392	Serpin	Defense mechanisms	9.35E-130
A3KMY8	KOG2024	Beta-Glucuronidase GUSB (glycosylhydrolase superfamily 2)	Carbohydrate transport and metabolism	1.82E-125
A4IFC0	---	no hits	no hits	---
A4IFR2	---	no hits	no hits	---
A5D7U1	---	no hits	no hits	---
A5D984	KOG2323	Pyruvate kinase	Carbohydrate transport and metabolism	0
A5PJL8	---	no hits	no hits	---
A5PKI3	---	no hits	no hits	---
A6BMK7	---	no hits	no hits	---
A6QLR7	KOG3867	Sulfatase	General function prediction only	3.12E-145
A6QLS9	KOG0078	GTP-binding protein SEC4, small G protein superfamily, and related Ras family GTP-binding proteins	Signal transduction mechanisms, Intracellular trafficking, secretion, and vesicular transport	2.34E-124
A6QLZ3	KOG3867	Sulfatase	General function prediction only	3.23E-161
A6QM01	KOG2233	Alpha-N-acetylglucosaminidase	Intracellular trafficking, secretion, and vesicular transport	0
A6QNJ8	KOG1066	Glucosidase II catalytic (alpha) subunit and related enzymes, glycosyl hydrolase family 31	Carbohydrate transport and metabolism, Cell wall/membrane/envelope biogenesis, Posttranslational modification, protein turnover, chaperones	0

A6QNL5	KOG0191	Thioredoxin/protein disulfide isomerase	Posttranslational modification, protein turnover, chaperones	1.27E-115
A6QNX2	KOG2183	Prolylcarboxypeptidase (angiotensinase C)	Posttranslational modification, protein turnover, chaperones, General function prediction only	0
A6QP36	KOG3839	Lectin VIP36, involved in the transport of glycoproteins carrying high mannose-type glycans	Intracellular trafficking, secretion, and vesicular transport	3.69E-168
A6QP39	---	no hits	no hits	---
A6QPK0	---	no hits	no hits	---
A6QPT4	KOG2408	Peroxidase/oxygenase	General function prediction only	0
A6QPZ4	KOG2392	Serpin	Defense mechanisms	7.31E-125
A6QQA8	KOG1731	FAD-dependent sulfhydryl oxidase/quiescin and related proteins	Cell cycle control, cell division, chromosome partitioning	0
A7E3W2	KOG4441	Proteins containing BTB/POZ and Kelch domains, involved in regulatory/signal transduction processes	Signal transduction mechanisms, General function prediction only	5.85E-17
A7MB70	KOG3611	Semaphorins	Signal transduction mechanisms	0
A7MBJ5	KOG1824	TATA-binding protein-interacting protein	General function prediction only	0
A8DC37	---	no hits	no hits	---
B0JYQ0	---	no hits	no hits	---
B5T255	---	no hits	no hits	---
B9TUD2	---	no hits	no hits	---
D4QBC5	---	no hits	no hits	---
D4QBD1	---	no hits	no hits	---
E1B6Z6	---	no hits	no hits	---
E1B748	KOG0104	Molecular chaperones GRP170/SIL1, HSP70 superfamily	Posttranslational modification, protein turnover, chaperones	0
E1B818	KOG3545	Olfactomedin and related extracellular matrix glycoproteins	Extracellular structures	3.40E-134
E1B8A0	KOG2679	Purple (tartrate-resistant) acid phosphatase	Posttranslational modification, protein turnover, chaperones	8.80E-147
E1B9P4	KOG1565	Gelatinase A and related matrix metalloproteases	Posttranslational modification, protein turnover, chaperones, Extracellular structures	3.95E-30
E1BBK6	KOG1214	Nidogen and related basement membrane protein proteins	Cell wall/membrane/envelope biogenesis, Extracellular structures	2.85E-20
E1BBU4	KOG3607	Meltrins, fertilins and related Zn-dependent metalloproteinases of the ADAMs family	Posttranslational modification, protein turnover, chaperones	0
E1BDF3	KOG1217	Fibrillins and related proteins containing Ca2+-binding EGF-like domains	Signal transduction mechanisms	5.11E-16
E1BDR2	---	no hits	no hits	---
E1BDY3	KOG3648	Golgi apparatus protein (cysteine-rich fibroblast growth factor receptor)	Intracellular trafficking, secretion, and vesicular transport	0

E1BG25	---	no hits	no hits	---
E1BH06	KOG1366	Alpha-macroglobulin	Posttranslational modification, protein turnover, chaperones	0
E1BHP0	KOG1959	Glycosyl hydrolase, family 38 - alpha-mannosidase	Carbohydrate transport and metabolism	0
E1BI28	---	no hits	no hits	---
E1BI74	---	no hits	no hits	---
E1BKX1	---	no hits	no hits	---
E1BKZ9	KOG3511	Sortilin and related receptors	General function prediction only	0
E1BLI4	---	no hits	no hits	---
E1BMJ0	KOG2392	Serpin	Defense mechanisms	4.56E-70
E1BN79	KOG4389	Acetylcholinesterase/Butyrylcholinesterase	Signal transduction mechanisms	8.37E-127
E1BNJ9	KOG3627	Trypsin	Amino acid transport and metabolism	2.97E-80
E1BQ21	---	no hits	no hits	---
F1MCF5	KOG1651	Glutathione peroxidase	Posttranslational modification, protein turnover, chaperones	2.78E-73
F1MD95	KOG1834	Calsyntenin	Extracellular structures	0
F1MDF2	---	no hits	no hits	---
F1MGN0	KOG3611	Semaphorins	Signal transduction mechanisms	0
F1MGQ1	---	no hits	no hits	---
F1MGU7	KOG2579	Ficolin and related extracellular proteins	General function prediction only	3.15E-105
F1MHF1	KOG2692	Sialyltransferase	Carbohydrate transport and metabolism	4.55E-34
F1MHR8	---	no hits	no hits	---
F1MHS5	---	no hits	no hits	---
F1MLR4	---	no hits	no hits	---
F1MLW2	KOG4160	BPI/LBP/CETP family protein	Defense mechanisms	6.80E-21
F1MLW8	---	no hits	no hits	---
F1MNJ4	---	no hits	no hits	---
F1MS23	---	no hits	no hits	---
F1MTI7	KOG3338	Divalent cation tolerance-related protein	Inorganic ion transport and metabolism	6.10E-68
F1MU34	KOG1046	Puromycin-sensitive aminopeptidase and related aminopeptidases	Amino acid transport and metabolism, Posttranslational modification, protein turnover, chaperones	0
F1MUB9	KOG3627	Trypsin	Amino acid transport and metabolism	2.16E-86
F1MUF4	KOG0496	Beta-galactosidase	Carbohydrate transport and metabolism	0

F1MWD3	KOG0357	Chaperonin complex component, TCP-1 epsilon subunit (CCT5)	Posttranslational modification, protein turnover, chaperones	0
F1MWI1	---	no hits	no hits	---
F1MWT0	KOG4342	Alpha-mannosidase	Carbohydrate transport and metabolism	0
F1MX50	KOG3374	Cellular repressor of transcription	Transcription	2.50E-101
F1MY12	KOG4745	Metalloproteinase inhibitor TIMP and related proteins	General function prediction only	8.22E-15
F1MYX5	KOG0046	Ca2+-binding actin-bundling protein (fimbrin/plastin), EF-Hand protein superfamily	Cytoskeleton	0
F1MZ40	KOG1214	Nidogen and related basement membrane protein proteins	Cell wall/membrane/envelope biogenesis, Extracellular structures	2.11E-20
F1MZJ5	KOG3607	Meltrins, fertilins and related Zn-dependent metalloproteinases of the ADAMs family	Posttranslational modification, protein turnover, chaperones	0
F1N0F2	---	no hits	no hits	---
F1N152	KOG1320	Serine protease	Posttranslational modification, protein turnover, chaperones	2.14E-106
F1N303	KOG3538	Disintegrin metalloproteinases with thrombospondin repeats	Posttranslational modification, protein turnover, chaperones	0
F1N3U5	KOG0806	Carbon-nitrogen hydrolase	Amino acid transport and metabolism	9.99E-131
F1N554	KOG3736	Polypeptide N-acetylgalactosaminyltransferase	Posttranslational modification, protein turnover, chaperones	0
F1N5H2	KOG3340	Alpha-L-fucosidase	Carbohydrate transport and metabolism	0
F1N5W4	KOG2645	Type I phosphodiesterase/nucleotide pyrophosphatase	General function prediction only	6.26E-174
F1N7D7	KOG3781	Dystroglycan	Extracellular structures	3.12E-180
F1N7F3	KOG1025	Epidermal growth factor receptor EGFR and related tyrosine kinases	Signal transduction mechanisms	0
G1CW25	---	no hits	no hits	---
G3MWX7	---	no hits	no hits	---
G3MX66	---	no hits	no hits	---
G3MZ03	KOG1928	Alpha-1,4-N-acetylglucosaminyltransferase	Carbohydrate transport and metabolism	1.00E-145
G3N0V0	---	no hits	no hits	---
G3N2D8	KOG2410	Gamma-glutamyltransferase	Amino acid transport and metabolism	0
G3X6G7	---	no hits	no hits	---
G5E589	KOG0179	20S proteasome, regulatory subunit beta type PSMB1/PRE7	Posttranslational modification, protein turnover, chaperones	1.85E-139
G5E5M8	KOG1959	Glycosyl hydrolase, family 38 - alpha-mannosidase	Carbohydrate transport and metabolism	0
G5E5T5	---	no hits	no hits	---
G5E604	---	no hits	no hits	---
H7BWW2	KOG2499	Beta-N-acetylhexosaminidase	Carbohydrate transport and metabolism	0

L8HKR7	---	no hits	no hits	---
L8HP94	---	no hits	no hits	---
L8HPF5	---	no hits	no hits	---
L8HZ66	KOG1367	3-phosphoglycerate kinase	Carbohydrate transport and metabolism	0
L8HZP9	KOG3690	Angiotensin I-converting enzymes - M2 family peptidases	Amino acid transport and metabolism	0
L8I064	KOG3867	Sulfatase	General function prediction only	1.57E-129
L8I2G8	KOG2392	Serpin	Defense mechanisms	1.15E-46
L8IAF4	---	no hits	no hits	---
L8IAH9	KOG1731	FAD-dependent sulfhydryl oxidase/quiescin and related proteins	Cell cycle control, cell division, chromosome partitioning	8.78E-166
L8IC40	---	no hits	no hits	---
L8ICQ2	KOG4419	5' nucleotidase	Nucleotide transport and metabolism	0
L8IEA1	KOG2499	Beta-N-acetylhexosaminidase	Carbohydrate transport and metabolism	0
L8IHR1	KOG1219	Uncharacterized conserved protein, contains laminin, cadherin and EGF domains	Signal transduction mechanisms	0
L8IQM7	KOG1219	Uncharacterized conserved protein, contains laminin, cadherin and EGF domains	Signal transduction mechanisms	6.49E-14
L8IVU7	KOG0358	Chaperonin complex component, TCP-1 delta subunit (CCT4)	Posttranslational modification, protein turnover, chaperones	0
L8IXW5	KOG1340	Prosaposin	Lipid transport and metabolism, Carbohydrate transport and metabolism	8.39E-59
L8J2B5	KOG3540	Beta amyloid precursor protein	General function prediction only	0
L8J3B9	KOG4237	Extracellular matrix protein slit, contains leucine-rich and EGF-like repeats	Extracellular structures, Signal transduction mechanisms	0
L8J3G6	KOG0819	Annexin	Intracellular trafficking, secretion, and vesicular transport	4.19E-162
M5FKI8	---	no hits	no hits	---
O46375	KOG3006	Transthyretin and related proteins	Lipid transport and metabolism	7.91E-59
O77588	KOG1971	Lysyl hydroxylase	Posttranslational modification, protein turnover, chaperones	0
P00517	KOG0616	cAMP-dependent protein kinase catalytic subunit (PKA)	Signal transduction mechanisms	0
P00570	KOG3079	Uridylate kinase/adenylate kinase	Nucleotide transport and metabolism	2.58E-104
P00669	---	no hits	no hits	---
P01045	---	no hits	no hits	---
P01888	---	no hits	no hits	---
P01966	---	no hits	no hits	---

P02584	KOG1755	Profilin	Cytoskeleton	1.60E-49
P02672	---	no hits	no hits	---
P02676	KOG2579	Ficolin and related extracellular proteins	General function prediction only	2.94E-114
P02769	---	no hits	no hits	---
P02784	---	no hits	no hits	---
P04557	---	no hits	no hits	---
P05307	KOG0190	Protein disulfide isomerase (prolyl 4-hydroxylase beta subunit)	Posttranslational modification, protein turnover, chaperones	0
P07456	---	no hits	no hits	---
P07688	KOG1543	Cysteine proteinase Cathepsin L	Posttranslational modification, protein turnover, chaperones	4.93E-72
P08037	KOG3916	UDP-Gal:glucosylceramide beta-1,4-galactosyltransferase	Carbohydrate transport and metabolism	0
P0C0S9	KOG1756	Histone 2A	Chromatin structure and dynamics	3.55E-73
P10096	KOG0657	Glyceraldehyde 3-phosphate dehydrogenase	Carbohydrate transport and metabolism	1.40E-180
P10152	---	no hits	no hits	---
P12763	---	no hits	no hits	---
P13600	---	no hits	no hits	---
P13696	KOG3346	Phosphatidylethanolamine binding protein	General function prediction only	2.23E-91
P14769	---	no hits	no hits	---
P15497	---	no hits	no hits	---
P16116	KOG1577	Aldo/keto reductase family proteins	General function prediction only	4.51E-154
P17697	---	no hits	no hits	---
P19120	KOG0101	Molecular chaperones HSP70/HSC70, HSP70 superfamily	Posttranslational modification, protein turnover, chaperones	0
P19879	KOG4237	Extracellular matrix protein slit, contains leucine-rich and EGF-like repeats	Extracellular structures, Signal transduction mechanisms	1.74E-13
P22226	---	no hits	no hits	---
P25326	KOG1543	Cysteine proteinase Cathepsin L	Posttranslational modification, protein turnover, chaperones	4.66E-111
P28291	---	no hits	no hits	---
P29392	---	no hits	no hits	---
P31096	---	no hits	no hits	---
P31976	KOG3529	Radixin, moesin and related proteins of the ERM family	General function prediction only	0
P34955	KOG2392	Serpin	Defense mechanisms	3.15E-126
P37141	KOG1651	Glutathione peroxidase	Posttranslational modification, protein turnover, chaperones	2.12E-74

P38657	KOG0190	Protein disulfide isomerase (prolyl 4-hydroxylase beta subunit)	Posttranslational modification, protein turnover, chaperones	0
P40682	KOG3868	Vacuolar H+-ATPase V0 sector, accessory subunit S1 (Ac45)	Energy production and conversion	1.95E-158
P45478	KOG2541	Palmitoyl protein thioesterase	Lipid transport and metabolism, Posttranslational modification, protein turnover, chaperones	2.67E-139
P52193	KOG0674	Calreticulin	Posttranslational modification, protein turnover, chaperones	0
P55206	---	no hits	no hits	---
P62803	KOG3467	Histone H4	Chromatin structure and dynamics	8.36E-56
P62935	KOG0865	Cyclophilin type peptidyl-prolyl cis-trans isomerase	Posttranslational modification, protein turnover, chaperones	7.79E-126
P62992	KOG0004	Ubiquitin/40S ribosomal protein S27a fusion	Translation, ribosomal structure and biogenesis	2.73E-90
P63103	KOG0841	Multifunctional chaperone (14-3-3 family)	Posttranslational modification, protein turnover, chaperones	1.77E-141
P63258	KOG0676	Actin and related proteins	Cytoskeleton	0
P68103	KOG0052	Translation elongation factor EF-1 alpha/Tu	Translation, ribosomal structure and biogenesis	0
P79345	KOG4063	Major epididymal secretory protein HE1	Function unknown	8.96E-61
P80025	KOG2408	Peroxidase/oxygenase	General function prediction only	0
P80311	KOG0880	Peptidyl-prolyl cis-trans isomerase	Posttranslational modification, protein turnover, chaperones	9.99E-128
P81019	---	no hits	no hits	---
P81134	KOG4737	ATPase membrane sector associated protein	Energy production and conversion	1.15E-168
P81187	KOG3627	Trypsin	Amino acid transport and metabolism	4.09E-34
P81265	---	no hits	no hits	---
P81425	KOG2100	Dipeptidyl aminopeptidase	Posttranslational modification, protein turnover, chaperones	0
P81947	KOG1376	Alpha tubulin	Cytoskeleton	0
P82292	---	no hits	no hits	---
Q08DW4	KOG3627	Trypsin	Amino acid transport and metabolism	1.02E-65
Q08E54	KOG3540	Beta amyloid precursor protein	General function prediction only	0
Q0IIH5	KOG3866	DNA-binding protein of the nucleobindin family	General function prediction only	0
Q0P569	KOG3866	DNA-binding protein of the nucleobindin family	General function prediction only	0
Q0V8B6	---	no hits	no hits	---
Q0V8R6	KOG2499	Beta-N-acetylhexosaminidase	Carbohydrate transport and metabolism	0
Q0VCQ9	KOG4223	Reticulocalbin, calumenin, DNA supercoiling factor, and related Ca2+-binding proteins of the CREC family (EF-Hand protein superfamily)	Signal transduction mechanisms, Intracellular trafficking, secretion, and vesicular transport	5.78E-117
Q0VCX2	KOG0100	Molecular chaperones GRP78/BiP/KAR2, HSP70 superfamily	Posttranslational modification, protein turnover, chaperones	0

Q0VD19	KOG3770	Acid sphingomyelinase and PHM5 phosphate metabolism protein	Lipid transport and metabolism	0
Q148N3	KOG1565	Gelatinase A and related matrix metalloproteases	Posttranslational modification, protein turnover, chaperones, Extracellular structures	5.01E-92
Q17QB3	---	no hits	no hits	---
Q17QC7	---	no hits	no hits	---
Q17QK3	KOG2195	Transferrin receptor and related proteins containing the protease-associated (PA) domain	Posttranslational modification, protein turnover, chaperones, Inorganic ion transport and metabolism, General function prediction only	1.24E-105
Q17QR9	KOG3555	Ca2+-binding proteoglycan Testican	General function prediction only	0
Q1JPB0	KOG2392	Serpin	Defense mechanisms	4.12E-143
Q1LZH9	KOG3731	Sulfatases	Carbohydrate transport and metabolism	0
Q1RMN0	KOG1593	Asparaginase	Amino acid transport and metabolism	4.02E-152
Q1RMN8	---	no hits	no hits	---
Q1ZYR0	---	no hits	no hits	---
Q28017	KOG3847	Phospholipase A2 (platelet-activating factor acetylhydrolase in humans)	Lipid transport and metabolism	0
Q28085	---	no hits	no hits	---
Q29443	---	no hits	no hits	---
Q29444	KOG2230	Predicted beta-mannosidase	Carbohydrate transport and metabolism	0
Q29451	KOG1959	Glycosyl hydrolase, family 38 - alpha-mannosidase	Carbohydrate transport and metabolism	0
Q2HJF0	---	no hits	no hits	---
Q2KHZ8	KOG2566	Beta-glucocerebrosidase	Carbohydrate transport and metabolism	0
Q2KIF2	KOG4194	Membrane glycoprotein LIG-1	Signal transduction mechanisms	6.01E-32
Q2KIM0	KOG3340	Alpha-L-fucosidase	Carbohydrate transport and metabolism	0
Q2KIY5	KOG3774	Uncharacterized conserved protein Lama	Signal transduction mechanisms	0
Q2KJF1	---	no hits	no hits	---
Q2LGB2	---	no hits	no hits	---
Q2NKZ9	KOG1283	Serine carboxypeptidases	Posttranslational modification, protein turnover, chaperones	0
Q2NL29	KOG0693	Myo-inositol-1-phosphate synthase	Lipid transport and metabolism	0
Q2T9S4	KOG2882	p-Nitrophenyl phosphatase	Inorganic ion transport and metabolism	1.76E-135
Q2TBU0	KOG3627	Trypsin	Amino acid transport and metabolism	2.77E-48
Q2TBX4	KOG0101	Molecular chaperones HSP70/HSC70, HSP70 superfamily	Posttranslational modification, protein turnover, chaperones	4.28E-133

Q2UVX4	KOG1366	Alpha-macroglobulin	Posttranslational modification, protein turnover, chaperones	0
Q32KV0	KOG0235	Phosphoglycerate mutase	Carbohydrate transport and metabolism	5.84E-108
Q32L40	KOG0360	Chaperonin complex component, TCP-1 alpha subunit (CCT1)	Posttranslational modification, protein turnover, chaperones	0
Q32L48	KOG1744	Histone H2B	Chromatin structure and dynamics	5.22E-58
Q32LF7	KOG3916	UDP-Gal:glucosylceramide beta-1,4-galactosyltransferase	Carbohydrate transport and metabolism	4.68E-151
Q32LQ0	KOG1046	Puromycin-sensitive aminopeptidase and related aminopeptidases	Amino acid transport and metabolism, Posttranslational modification, protein turnover, chaperones	0
Q3B7N2	KOG0035	Ca2+-binding actin-bundling protein (actinin), alpha chain (EF-Hand protein superfamily)	Cytoskeleton	0
Q3MHM5	KOG1375	Beta tubulin	Cytoskeleton	0
Q3MHN5	---	no hits	no hits	---
Q3MHX6	KOG3394	Protein OS-9	General function prediction only	7.35E-151
Q3MI05	KOG1282	Serine carboxypeptidases (lysosomal cathepsin A)	Amino acid transport and metabolism, Posttranslational modification, protein turnover, chaperones	0
Q3SX14	KOG0443	Actin regulatory proteins (gelsolin/villin family)	Cytoskeleton	0
Q3SX46	KOG2246	Galactosyltransferases	Carbohydrate transport and metabolism	1.38E-98
Q3SYR8	---	no hits	no hits	---
Q3SYW2	KOG3627	Trypsin	Amino acid transport and metabolism	1.54E-30
Q3SZB7	KOG1458	Fructose-1,6-bisphosphatase	Carbohydrate transport and metabolism	0
Q3SZV7	KOG1565	Gelatinase A and related matrix metalloproteases	Posttranslational modification, protein turnover, chaperones, Extracellular structures	6.92E-56
Q3T010	KOG3346	Phosphatidylethanolamine binding protein	General function prediction only	3.25E-57
Q3T052	KOG2353	L-type voltage-dependent Ca2+ channel, alpha2/delta subunit	Inorganic ion transport and metabolism, Signal transduction mechanisms	1.48E-12
Q3T0A3	KOG3627	Trypsin	Amino acid transport and metabolism	4.29E-71
Q3T0K2	KOG0364	Chaperonin complex component, TCP-1 gamma subunit (CCT3)	Posttranslational modification, protein turnover, chaperones	0
Q3T0Z0	KOG4802	Adhesion-type protein	Extracellular structures	9.13E-11
Q3T149	KOG3591	Alpha crystallins	Posttranslational modification, protein turnover, chaperones	3.30E-63
Q3ZBD7	KOG2446	Glucose-6-phosphate isomerase	Carbohydrate transport and metabolism	0
Q3ZBH0	KOG0363	Chaperonin complex component, TCP-1 beta subunit (CCT2)	Posttranslational modification, protein turnover, chaperones	0
Q3ZBM0	---	no hits	no hits	---
Q3ZBS7	KOG1565	Gelatinase A and related matrix metalloproteases	Posttranslational modification, protein turnover, chaperones, Extracellular structures	6.82E-42

Q3ZBZ1	KOG4251	Calcium binding protein	General function prediction only	0
Q3ZC64	KOG3858	Ephrin, ligand for Eph receptor tyrosine kinase	Signal transduction mechanisms	4.38E-62
Q3ZCH5	---	no hits	no hits	---
Q3ZCI9	KOG0362	Chaperonin complex component, TCP-1 theta subunit (CCT8)	Posttranslational modification, protein turnover, chaperones	0
Q4R0H2	---	no hits	no hits	---
Q4W1E4	KOG3690	Angiotensin I-converting enzymes - M2 family peptidases	Amino acid transport and metabolism	0
Q58CQ9	KOG0806	Carbon-nitrogen hydrolase	Amino acid transport and metabolism	1.19E-130
Q58DP6	---	no hits	no hits	---
Q5DPW9	---	no hits	no hits	---
Q5E946	KOG2764	Putative transcriptional regulator DJ-1	General function prediction only, Defense mechanisms	2.57E-83
Q5E998	KOG1543	Cysteine proteinase Cathepsin L	Posttranslational modification, protein turnover, chaperones	1.64E-117
Q5E9B1	KOG1495	Lactate dehydrogenase	Energy production and conversion	0
Q5E9I4	KOG1413	N-acetylglucosaminyltransferase I	Carbohydrate transport and metabolism	0
Q5EA54	KOG0471	Alpha-amylase	Carbohydrate transport and metabolism	3.58E-76
Q5EA62	KOG1214	Nidogen and related basement membrane protein proteins	Cell wall/membrane/envelope biogenesis, Extracellular structures	1.86E-20
Q5W5U3	KOG1369	Hexokinase	Carbohydrate transport and metabolism	0
Q6R8F2	KOG4289	Cadherin EGF LAG seven-pass G-type receptor	Signal transduction mechanisms	9.65E-45
Q6T182	---	no hits	no hits	---
Q76LV2	KOG0019	Molecular chaperone (HSP90 family)	Posttranslational modification, protein turnover, chaperones	0
Q7SIH1	KOG1366	Alpha-macroglobulin	Posttranslational modification, protein turnover, chaperones	0
Q7YRE5	---	no hits	no hits	---
Q7YRQ8	KOG4295	Serine proteinase inhibitor (KU family)	Posttranslational modification, protein turnover, chaperones	9.96E-17
Q863C3	---	no hits	no hits	---
Q863K8	KOG1476	Beta-1,3-glucuronyltransferase B3GAT1/SQV-8	Posttranslational modification, protein turnover, chaperones	2.52E-133
Q8HZY1	KOG2392	Serpin	Defense mechanisms	1.59E-116
Q8SPU5	---	no hits	no hits	---
Q95114	KOG1094	Discoidin domain receptor DDR1	Signal transduction mechanisms	4.77E-25
Q95121	KOG2392	Serpin	Defense mechanisms	1.42E-85
Q95M12	KOG1348	Asparaginyl peptidases	Posttranslational modification, protein turnover, chaperones	0
Q95M18	KOG0020	Endoplasmic reticulum glucose-regulated protein (GRP94/endoplasmin), HSP90 family	Posttranslational modification, protein turnover, chaperones	0

Q9BEG4	KOG2692	Sialyltransferase	Carbohydrate transport and metabolism	1.70E-99
Q9BGI1	KOG0541	Alkyl hydroperoxide reductase/peroxiredoxin	Posttranslational modification, protein turnover, chaperones	1.09E-73
Q9BGI2	KOG0852	Alkyl hydroperoxide reductase, thiol specific antioxidant and related enzymes	Posttranslational modification, protein turnover, chaperones	1.02E-130
Q9BGU5	KOG1339	Aspartyl protease	Posttranslational modification, protein turnover, chaperones	3.29E-97
Q9GLE5	KOG1565	Gelatinase A and related matrix metalloproteases	Posttranslational modification, protein turnover, chaperones, Extracellular structures	7.45E-145
Q9MYM4	KOG1065	Maltase glucoamylase and related hydrolases, glycosyl hydrolase family 31	Carbohydrate transport and metabolism	0
Q9N2I2	KOG2392	Serpin	Defense mechanisms	3.61E-123
Q9XSC9	---	no hits	no hits	---
Q9XSJ4	KOG2670	Enolase	Carbohydrate transport and metabolism	0
U5Y6U2	---	no hits	no hits	---
W0UVF3	---	no hits	no hits	---
W5NTH3	KOG4193	G protein-coupled receptors	Signal transduction mechanisms	3.91E-128
W5NXW9	---	no hits	no hits	---
W5P198	---	no hits	no hits	---
W5P1A8	KOG0200	Fibroblast/platelet-derived growth factor receptor and related receptor tyrosine kinases	Signal transduction mechanisms	2.56E-145
W5Q3V3	---	no hits	no hits	---
